# Decoupled Spatiotemporal Learning for Multi-Location Beam Response Reconstruction from Sparse Deflection Measurements

**DOI:** 10.3390/s26206635

**Published:** 2026-10-20

**Authors:** Zhenyi Luo, Fei Yan, Yu Wen, Yan Geng, Dongmei Yan

**Affiliations:** 1 Hunan Provincial Key Laboratory of Structural Engineering for Wind Resistant and Vibration Control, Hunan University of Science and Technology, Xiangtan 411201, China; 2 Institute of Protected Agriculture, Academy of Agricultural Planning and Engineering, MARA, Beijing 100125, China; 3 Central Research Institute of Building and Construction, MCC Group Ltd., Beijing 100089, China

**Keywords:** structural health monitoring, multi-location response reconstruction, virtual sensing, physics-informed neural networks, spatiotemporal decoupling, simulation-to-real transfer learning

## Abstract

Reconstructing continuous deflection and stress fields from sparse deflection measurements is challenging because the two responses have different spatial distributions and stress depends on deflection curvature. Limited experimental data further constrain model training and cross-domain adaptation. We present a decoupled spatiotemporal physics-informed neural network (DS-PINN) for reconstructing low-frequency elastic responses. The method combines latent temporal integration, dual-response spatial representation and simulation-to-experiment adaptation. Rather than representing the full spatiotemporal field with a single coordinate network, DS-PINN evolves latent coordinates through a Newmark-β integration operator. A shared sinusoidal representation network (SIREN) backbone extracts continuous spatial features, and two response-specific regression heads produce separate bases for deflection and stress. The model was pretrained on 1800 simulated records and fine-tuned on 16 measured records, while 200 simulated and 5 measured records were used for validation. Only three deflection channels were supplied as inputs. Across four independent 55 s test records, DS-PINN achieved Pearson correlation coefficients of 0.9323 for deflection and 0.9093 for stress. The corresponding normalized root-mean-square error (NRMSE) values were 7.94% and 7.59%. Spatial NRMSE values remained below 6% at all 11 evaluation locations. Compared with the closest end-to-end baseline, a temporal convolutional network, DS-PINN reduced deflection and stress NRMSE by approximately 45.0% and 45.9%, respectively. Ablation tests identified distinct roles for Newmark-based latent dynamics, temporal adaptation, magnitude correction, and spatial-basis consistency. These results support a simulation-assisted approach to multi-location virtual sensing of low-frequency beam responses from sparse deflection measurements and limited experimental data.

## 1. Introduction

Structural health monitoring (SHM) acquires the in-service responses of structures through deployed sensors [1]. Because the number and placement of measurement points are constrained, discrete observations cannot fully capture continuous spatiotemporal variations in structural responses. For low-frequency elastic vibration, deflection primarily reflects the global deformation of a structure, whereas stress characterises local loading and is more sensitive to changes in curvature. Their continuous distributions therefore provide complementary information for structural condition assessment. To extend spatial coverage, Shao et al. [2] combined object segmentation, background removal, and monocular depth estimation to obtain full-field out-of-plane vibration displacements. Xu et al. [3] integrated distributed fibre-optic strain measurements with sparse pointwise deflection observations to extend deflection estimates at critical sections to continuous locations along a main girder. These methods improve the spatial coverage of displacement fields, but remain constrained by observation conditions, sensor layouts, and transformation models. Stress measurements also continue to rely mainly on local strain sensing. Inferring responses at uninstrumented locations from limited observations is therefore an important problem in full-field virtual sensing for structures [4,5].

Existing methods for structural-response reconstruction can be broadly classified as model-driven or data-driven. Model-driven methods generally use state-space equations or finite element models (FEMs) to establish relationships between measured and unmeasured responses. He et al. [6] fused displacement obtained by integrating acceleration with displacement inferred from strain, thereby reconstructing dynamic and low-frequency quasi-static displacements under moving loads. Zhu et al. [7] used a unified linear input and state estimator to integrate heterogeneous sensor information under unknown loading and rank-deficient conditions. Narouie et al. [8] combined model priors with sparse observations through a statistical finite element method to infer displacement fields in unmeasured regions. These methods have a clear mechanical basis, but their reconstruction accuracy depends on the reliability of the structural model, boundary conditions, and sensor information.

Data-driven methods instead learn nonlinear mappings between measurement points directly from monitoring data and exploit spatiotemporal correlations among channels to recover missing responses [9]. Jiang et al. [10] formulated response reconstruction as sequence generation. Their sequence-to-sequence model, based on bidirectional gated recurrent units and a soft-attention mechanism, exploited latent spatiotemporal correlations to reconstruct responses under low-amplitude ambient excitation. Subsequent studies used self-attention-enhanced generative adversarial networks to learn correlations among responses at multiple measurement points [11]. A temporal convolutional network (TCN) with a joint time–frequency loss simultaneously constrained time-domain errors and frequency-domain information [12]. A long short-term memory (LSTM) network combined with the short-time Fourier transform improved the robustness of response estimation under noisy conditions [13]. However, these models usually require representative measured samples. Their output locations are also commonly tied to predefined channels, which prevents them from defining a continuous response function of spatial coordinates. Moreover, joint representations remain underexplored for responses such as deflection and stress, which are driven by the same dynamic process but have different spatial distributions.

Physics-informed neural networks (PINNs) incorporate partial differential equation (PDE) residuals, boundary conditions, and observational data into training, providing a new route to continuous-field inference from sparse observations [14,15]. Haghighat et al. [16] embedded momentum balance and constitutive relations in neural networks and used multiple networks to represent different field variables, enabling the joint solution of displacement and stress fields in solid mechanics. Go et al. [17] used sparse local strain measurements and spatial coordinates as inputs to infer full-field displacement and stress in a two-dimensional linear elastic problem, demonstrating the feasibility of PINNs for multi-field virtual sensing. For beam structures, Al-Adly and Kripakaran [18] progressively incorporated position, vehicle passage time, and load magnitude into a PINN to predict deflection, strain, and internal forces at uninstrumented locations. Liang et al. [19] transformed the governing equations into the frequency domain to mitigate spectral bias in multifrequency structural-response calculations.

Including governing equations in a loss function does not, however, guarantee stable training. Different loss components may converge at substantially different rates [20]. For time-dependent problems, a PINN may also converge to an incorrect solution if training does not reflect the spatiotemporal causality of the physical system [21]. For an Euler–Bernoulli beam, a single coordinate network must simultaneously process temporal and spatial derivatives of different orders, while stress depends on deflection curvature. Under sparse observations, this coupling of derivatives can make training more difficult and propagate local deflection errors through spatial differentiation into the reconstructed stress field.

Limited experimental data present another challenge for full-field reconstruction. Li et al. [22] used a convolutional autoencoder to estimate equivalent loads and calculated full-field structural responses from an impulse-response matrix generated by an FEM. Multifidelity learning reduces the demand for costly high-fidelity samples by combining data of different fidelities [23]. Related studies have treated approximate governing equations as low-fidelity information, integrated them with sparse experimental data through multifidelity PINNs and transfer learning [24], and reused models pretrained under simplified loading conditions to accommodate different structures and loading scenarios [25]. Chen et al. [26] proposed a two-stage variable-fidelity data-fusion method. They first pretrained a convolutional autoencoder on numerous simulated stress fields and then jointly updated the decoder using simulated and measured data, enabling full-field stress reconstruction from only a few sensors. Simulation data can therefore partly offset the limited availability of measured samples [27]. Related approaches represent structural dynamics in complementary ways. Lee and Carlberg [28] used autoencoders to define nonlinear manifolds for projection-based model reduction. DeepONet [29] learns mappings between functions by encoding input functions and output coordinates separately. Sharma and Kramer [30] learned reduced-order operators while preserving Lagrangian structure. These approaches support reduced representations, coordinate-dependent outputs, and physics-guided dynamics. Here, the aim is to combine shared temporal dynamics with separate spatial representations of deflection and stress for sparse beam sensing, while adapting from simulated to measured responses.

This study therefore proposes a decoupled spatiotemporal physics-informed neural network (DS-PINN) that models latent-space temporal evolution and continuous spatial distributions separately. The method uses a latent-space recurrence module to model low-frequency responses over long durations. A spatial module driven by shared latent coordinates represents continuous deflection and stress fields. Simulation pretraining is followed by physical-domain fine-tuning, allowing the model to adapt to measured responses when only a small experimental dataset is available. DS-PINN separates temporal evolution from coordinate-dependent spatial representation, unlike direct sequence-to-channel models. It differs from a PINN built on a single coordinate network by using Newmark integration and response-specific spatial bases. During inference, it predicts stress without differentiating the reconstructed deflection.

The remainder is organized as follows. Section 2 defines the problem and describes data generation and input processing. Section 3 presents the DS-PINN architecture and training procedure. Section 4 evaluates and analyses model performance. Section 5 presents the conclusions.

## 2. Experiments and Simulations

We evaluated DS-PINN using the low-frequency vertical bending response of a beam under sparse observations and limited measured data. This section defines the reconstruction problem and describes the experiment, simulation, evaluation metrics, and signal preprocessing.

### 2.1. Problem Formulation and Experimental Setup

#### 2.1.1. Response Equations and Reconstruction Problem

Within the linear elastic range and under the small-deformation assumption, the forced vertical bending vibration of the test beam can be approximated by the one-dimensional Euler–Bernoulli beam model:

(1) $$\mu \frac{\partial^{2}u(z,t)}{\partial t^{2}}+c\frac{\partial u(z,t)}{\partial t}+\mathrm{EI}\frac{\partial^{4}u(z,t)}{\partial z^{4}}=q(z,t)$$

where *μ* is the mass per unit length, *u*(*z*,*t*) is the vertical deflection, *c* is the damping coefficient, *E* is the elastic modulus, *I* is the second moment of area, and *q*(*z*,*t*) is the vertical external load per unit length.

Under the plane-section assumption, the longitudinal normal stress is related to deflection by:

(2) $$\sigma (z,t)=-\mathrm{Ey}\frac{\partial^{2}u(z,t)}{\partial z^{2}}$$

where *y* is the coordinate of the evaluation point relative to the neutral axis and *σ*(*z*,*t*) is the longitudinal normal stress.

The physical-domain tests considered here were dominated by low-frequency vertical excitation. When the excitation angular frequency *ω* is substantially lower than the first vertical-bending natural angular frequency *ω*_1_, the influence of the inertia term relative to the stiffness term scales as ${\left(\omega /\omega_{1}\right)}^{2}$, and the structural response becomes stiffness dominated. Under this condition, deflection is governed mainly by low-frequency global deformation, while higher-order modal contributions are relatively weak. Because the number and locations of sensors are limited, sparse displacement measurements can capture the overall deformation trend but constrain local curvature poorly in unobserved regions. Equation (2) shows that stress is determined by the second spatial derivative of deflection and is therefore more sensitive to local errors in the deflection field. Reconstructing stress in unobserved regions is consequently more difficult than reconstructing deflection.

In a PINN based on a single coordinate network, the network must simultaneously evaluate the low-order temporal derivatives and high-order spatial derivatives in Equation (1), while stress reconstruction also requires the second spatial derivative in Equation (2). Joint optimization of derivatives of different orders may complicate training and propagate deflection errors into the reconstructed stress field. The central problem addressed here is therefore the multi-location reconstruction of deflection and stress under low-frequency excitation, sparse observations, and limited measured data.

#### 2.1.2. Experiment and Modelling

**Figure 1:**
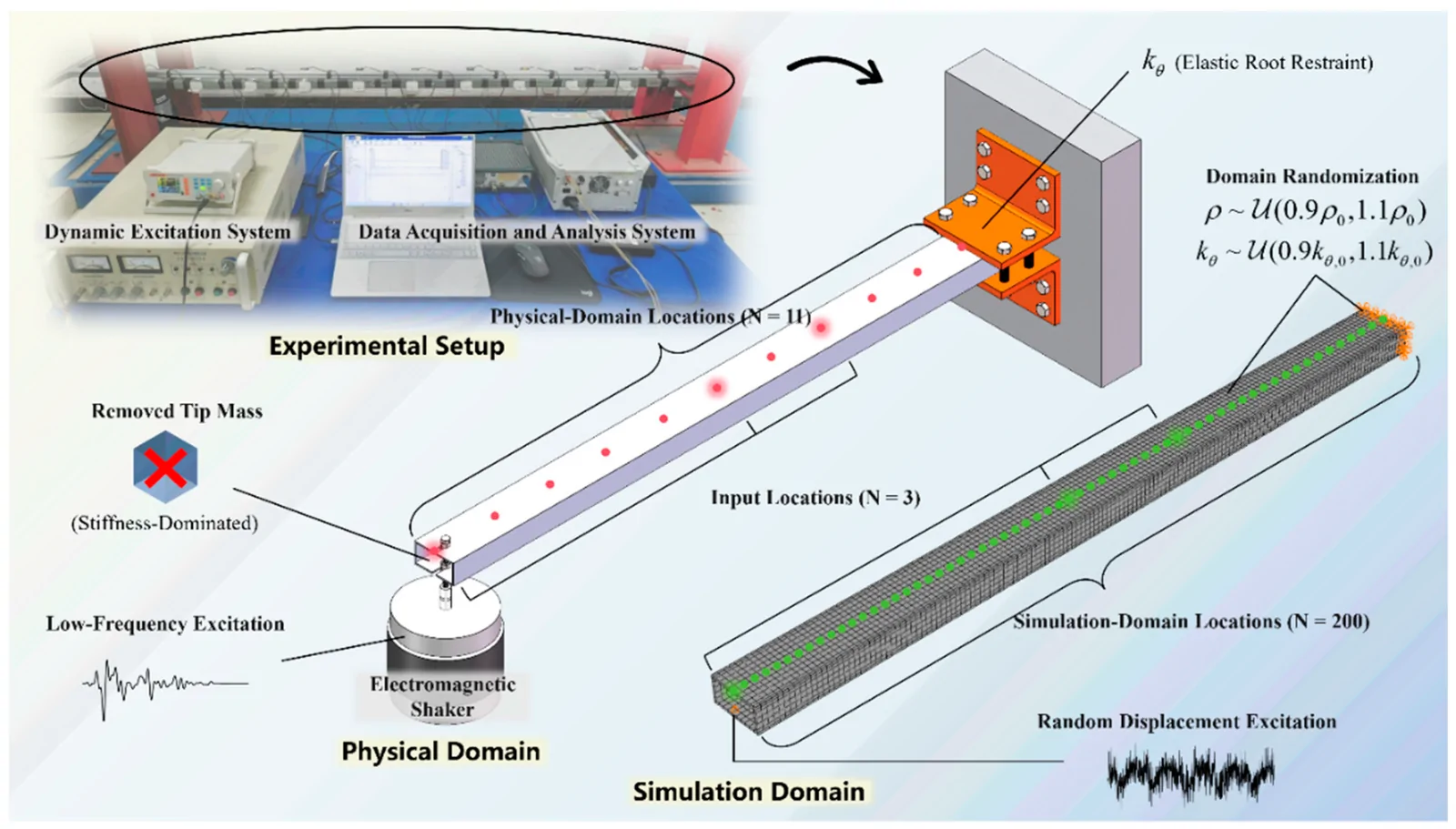
Experimental setup and finite element model.

A physical test rig and its corresponding finite element model were established, as shown in Figure 1. In the physical experiment, the added tip mass was removed and an electromagnetic shaker was connected through a hinge. The calculated first vertical-bending natural frequency for this configuration was 21.2 Hz. The excitation frequencies used in the tests were lower than this frequency, thereby maintaining a stiffness-dominated response. The FEM was constructed from the geometry and material properties of the test beam. Its main parameters are listed in Table 1. Four-node, reduced-integration S4R shell elements were used with a global mesh size of 25.0 mm to calculate vertical deflection and cross-sectional stress in the main beam.

**Figure 2:**
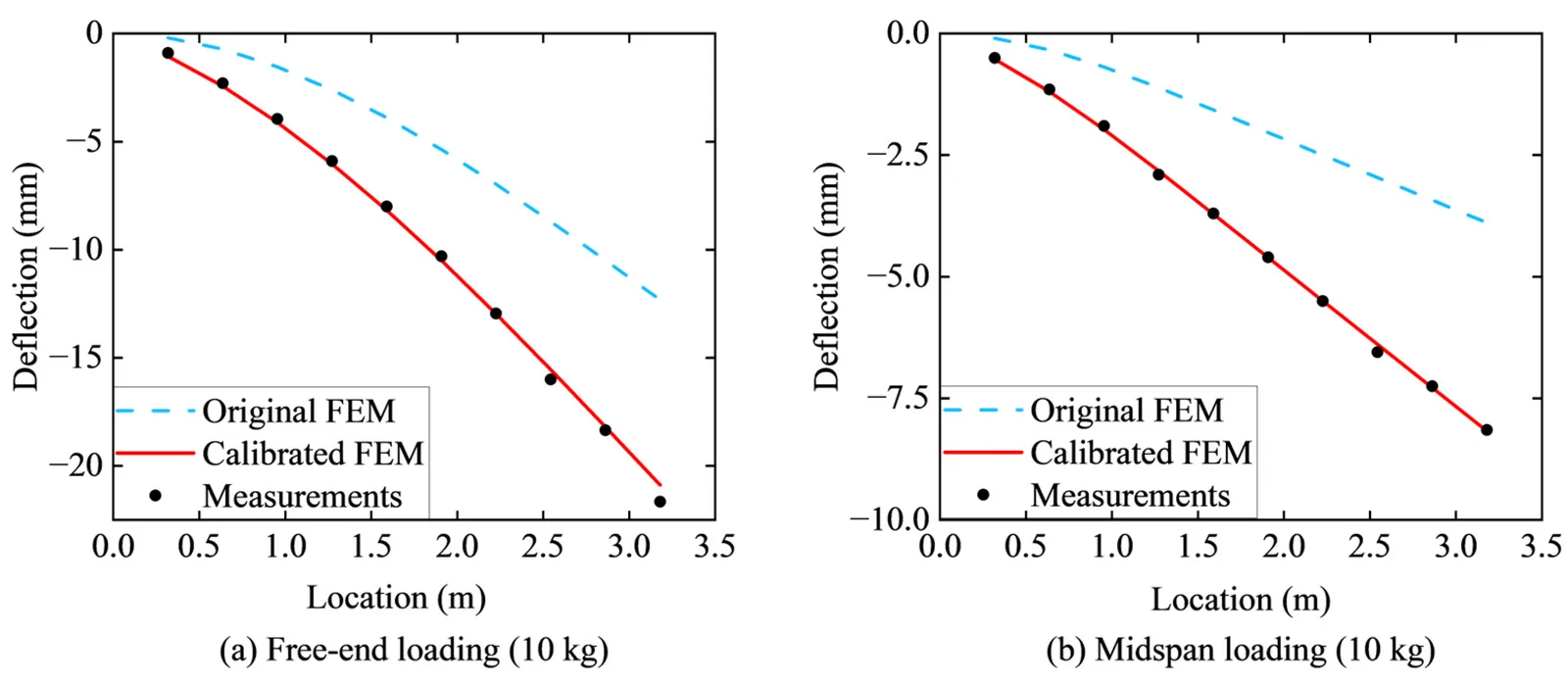
Measured static deflections compared with simulation results.

The root rotational stiffness was calibrated using static loading tests conducted after the shaker was removed from the beam. Masses of 5 and 10 kg were applied separately at the cantilever midspan and free end. Each loading case was repeated three times. The mean responses yielded a calibrated root rotational stiffness of *k_θ_*_,0_ = 1.13 × 10^8^ N ⋅ mm/rad. Figure 2 compares the measured deflection profiles with FEM predictions before and after calibration. Including root flexibility improved agreement along the beam.

For the 10 kg free-end loading case used in calibration, the mean measured free-end deflection was −21.65 mm, compared with −12.32 mm for the ideally clamped model and −20.88 mm after accounting for root flexibility. The absolute deviation decreased from 9.33 to 0.77 mm. In the FEM, the root cross-section was kinematically coupled to a reference point. Rotation about the principal bending axis was released and constrained by a rotational spring with the calibrated stiffness. The cross-section at the excitation end was likewise coupled to a reference point for application of the prescribed vertical displacement. Density and root rotational stiffness were varied to represent changes in inertia scale and boundary flexibility during pretraining. Both parameters were sampled independently from uniform distributions within ±10% of their nominal values for local data augmentation. All other structural parameters were held fixed: *ρ*~*U*(0.9*ρ*_0_,1.1*ρ*_0_) and *k_θ_*~*U*(0.9*k_θ_*_,0_,1.1*k_θ_*_,0_).

Under low-frequency, small-amplitude vibration, structural acceleration was weak. Conventional accelerometers therefore had a low signal-to-noise ratio and were susceptible to drift. High-precision laser displacement sensors were used to measure vertical deflection, while strain gauges were installed at corresponding positions. The acquisition and analysis software converted the strain signals and directly output longitudinal stress. Eleven measurement locations were uniformly distributed along the centreline of the upper surface of the main beam. Vertical deflection and longitudinal stress were acquired synchronously at all 11 locations, producing 22 response channels. Only three vertical-deflection channels, located at the excitation end, midspan, and 3*L*/10 from the root, were used as model inputs. Deflection and stress at all 11 locations served as labels for physical-domain fine-tuning and testing. Twenty-five dynamic-response records were acquired. Each record was 60 s long and sampled at 100 Hz. Section 3.3 describes the data partition.

### 2.2. Excitation and Simulation

The energy of the measured responses was concentrated mainly at low frequencies, and their spectral composition was relatively simple. For simulation pretraining, 2000 random displacement excitations were generated to cover the experimentally relevant low-frequency range and excite the first vertical-bending mode. Each signal was 60 s long, sampled at 100 Hz, and assigned a randomly selected peak amplitude between 1 and 20 mm.

**Figure 3:**
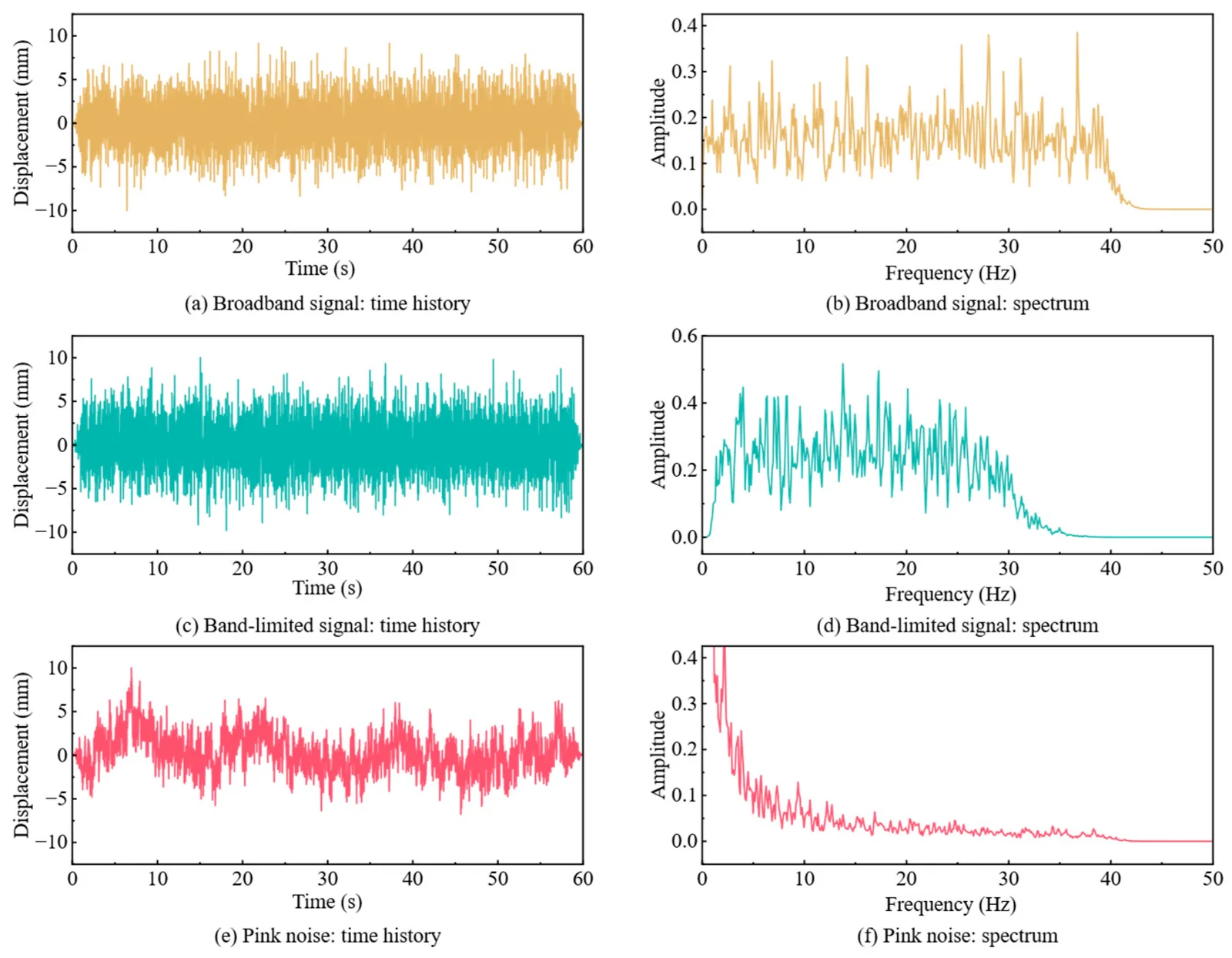
Time–frequency characteristics of simulation excitations.

As shown in Figure 3, the simulated excitations comprised 800 broadband Gaussian random signals, 800 band-limited random signals between 1 and 30 Hz, and 400 pink-noise signals. The broadband signals covered the principal frequency range below 40 Hz. The band-limited signals covered the 21.2 Hz first vertical-bending mode, and the pink-noise signals added conditions with a larger proportion of low-frequency energy. According to the Nyquist–Shannon sampling theorem, a sampling frequency of 100 Hz gives a maximum alias-free frequency of 50 Hz. All signals were therefore processed using a sixth-order Butterworth low-pass filter with a 40 Hz cut-off to suppress components near the sampling limit. A Tukey window with *α* = 0.05 was then applied to ensure temporal smoothness. Finally, each signal was amplitude-normalized and rescaled to its target peak value.

Finite element solutions were obtained using the Abaqus implicit dynamic procedure. The prescribed vertical displacement history was applied at the reference point of the excitation end. Each analysis lasted 60 s, with an initial time increment of 0.001 s, a maximum time increment of 0.01 s, and a maximum of 200,000 increments. Because the implicit integration procedure did not produce equally spaced output times, all responses were resampled to 100 Hz by linear interpolation during post-processing, giving 6001 time points between 0 and 60 s.

Vertical deflection and longitudinal stress were extracted along the centreline of the upper surface of the main beam, and stresses from adjacent elements were averaged at the nodes. Linear interpolation along the beam axis then generated deflection and stress histories at 200 equally spaced positions. As in the physical domain, the three vertical-deflection channels at the excitation end, midspan, and 3*L*/10 from the root were used as observation inputs. The interpolated deflection and stress histories at 200 positions served as supervised data for simulation pretraining. Section 3.2 describes the data partition.

### 2.3. Evaluation Metrics

We evaluated signal processing and response reconstruction using four metrics. The Pearson correlation coefficient (PCC) measured waveform correlation between reconstructed and reference responses. The normalized root-mean-square error (NRMSE) measured overall error. Peak error (PeakErr) measured the accuracy of reconstructed absolute peaks. Dominant-frequency amplitude error (FreqErr) measured amplitude deviation at the dominant frequency of the reference response. These metrics were calculated as follows:

(3) $$\mathrm{PCC}=\frac{1}{N_{\mathrm{valid}}}\sum_{j=1}^{N_{\mathrm{valid}}}\frac{\sum_{i=1}^{T}\left(y_{\mathrm{pred},i,j}-{\bar{y}}_{\mathrm{pred},j})(y_{\mathrm{gt},i,j}-{\bar{y}}_{\mathrm{gt},j}\right)}{\sqrt{\sum_{i=1}^{T}{\left(y_{\mathrm{pred},i,j}-{\bar{y}}_{\mathrm{pred},j}\right)}^{2}}\sqrt{\sum_{i=1}^{T}{\left(y_{\mathrm{gt},i,j}-{\bar{y}}_{\mathrm{gt},j}\right)}^{2}}}$$

(4) $$\mathrm{NRMSE}=\frac{1}{N_{\mathrm{valid}}}\sum_{j=1}^{N_{\mathrm{valid}}}\frac{\sqrt{\frac{1}{T}\sum_{i=1}^{T}{\left(y_{\mathrm{pred},i,j}-y_{\mathrm{gt},i,j}\right)}^{2}}}{\max_{1\leq i\leq T}(|y_{\mathrm{gt},i,j}|)}\times 100\%$$

(5) $$\mathrm{PeakErr}=\frac{1}{N_{\mathrm{valid}}}\sum_{j=1}^{N_{\mathrm{valid}}}\frac{\left|\max_{1\leq i\leq T}(|y_{\mathrm{pred},i,j}|)-\max_{1\leq i\leq T}(|y_{\mathrm{gt},i,j}|)\right|}{\max_{1\leq i\leq T}(|y_{\mathrm{gt},i,j}|)}\times 100\%$$

(6) $$\mathrm{FreqErr}=\frac{1}{N_{\mathrm{valid}}}\sum_{j=1}^{N_{\mathrm{valid}}}\frac{\left|A_{\mathrm{pred},j}^{f_{d,j}}-A_{\mathrm{gt},j}^{f_{d,j}}\right|}{\max_{1\leq k\leq N_{\mathrm{valid}}}(A_{\mathrm{gt},k}^{f_{d,k}})}\times 100\%$$

where *y*_pred,*i*,*j*_ and *y*_gt,*i*,*j*_ denote the predicted and measured responses, respectively, at valid measurement point *j* and discrete time step *i*(*i* = 1,…,*T*). *T* is the number of sampled time steps. ${\bar{y}}_{\mathrm{pred},j}$ and ${\bar{y}}_{\mathrm{gt},j}$ are the temporal means of the corresponding series at point *j*. *N*_valid_ is the number of valid measurement points. A point was excluded if the temporal variance of its reference response was no greater than 10^−6^, thereby avoiding numerical instability during normalization. max_1≤*i*≤*T*_(| ⋅ |) denotes the absolute temporal peak. *f*_d,*j*_ is the dominant frequency after removal of the direct-current component at point *j*. $A_{\mathrm{pred},j}^{f_{d,j}}$ and $A_{\mathrm{gt},j}^{f_{d,j}}$ are the predicted and measured amplitudes at this dominant frequency. $\max_{1\leq k\leq N_{\mathrm{valid}}}(A_{\mathrm{gt},k}^{f_{d,k}})$ is the maximum measured dominant-frequency amplitude among all valid points.

### 2.4. Input Preprocessing

**Figure 4:**
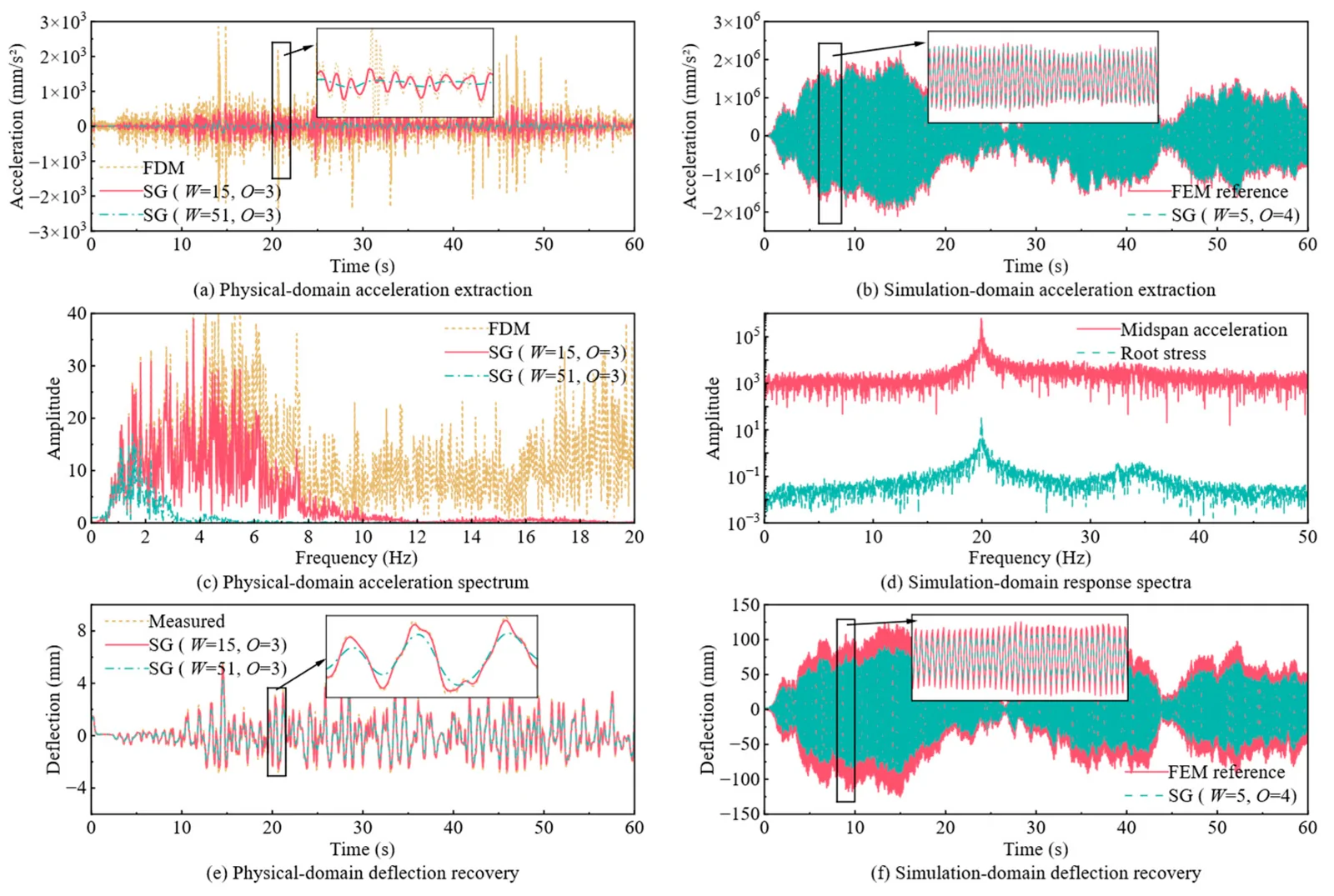
Signal processing in the physical and simulation domains.

Dynamic feature extraction and temporal evolution require equivalent acceleration. Although the laser displacement sensors provided deflection responses with a high signal-to-noise ratio, the measured signals still contained noise. Direct second-order numerical differentiation using the finite difference method (FDM) substantially amplified high-frequency noise, as shown in Figure 4a,c, and reduced the accuracy of the equivalent-acceleration estimates.

Savitzky–Golay (SG) filtering was therefore used for denoising and acceleration extraction in the physical domain. The window length and polynomial order were set to *W* = 15 and *O* = 3, respectively. The SG method fitted a polynomial within each local window and evaluated its second analytical derivative, thereby suppressing high-frequency noise while retaining the dominant response band, as shown in Figure 4c. To determine whether the differentiated signal retained the principal waveform of the original deflection, equivalent acceleration was integrated twice using the trapezoidal rule, and the integration trend was removed to obtain recovered deflection. This operation was used only to validate the SG parameters and was not part of the model calculation. Figure 4e shows that a longer window (*W* = 51) oversmoothed local waveform features, whereas the shorter window (*W* = 15) preserved them. The recovered and measured deflections were consequently in close agreement.

**Figure 5:**
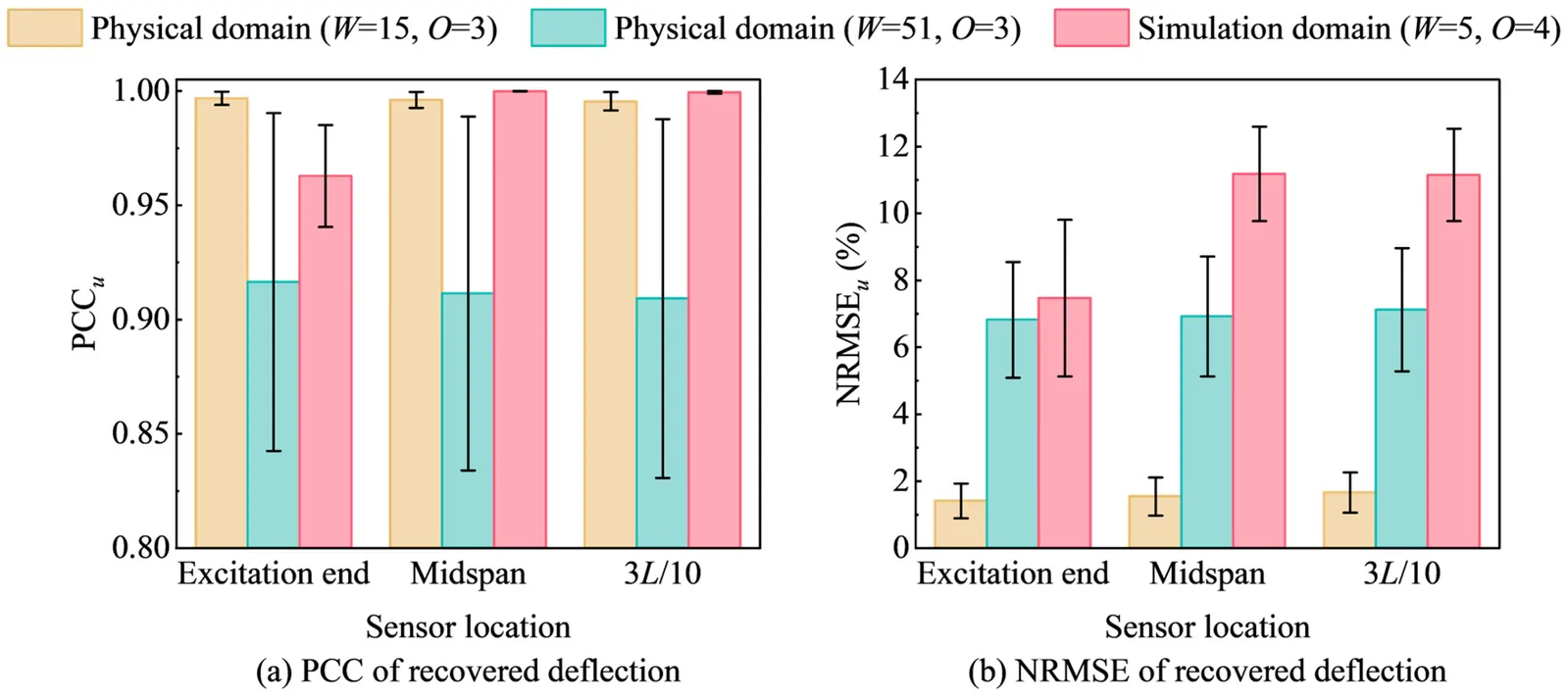
Evaluation of recovered deflection in the physical and simulation domains.

The simulated data contained no measurement noise, so the SG parameters were set to *W* = 5 and *O* = 4. This configuration corresponds to a five-point, fourth-order central difference and reduces the effect of second differentiation on the amplitudes of the dominant frequency components. Figure 4b shows that the SG-derived acceleration was consistent with the output from the finite element solver. As shown in Figure 4d, the midspan acceleration had a pronounced peak near 21.2 Hz, corresponding to the first vertical-bending mode of the main beam. The root stress retained a marked response near 34.5 Hz, indicating that stress contained appreciable high-frequency dynamic components. Figure 4 presents representative processed samples. The stability of the selected parameters across all samples was further assessed using the PCC and NRMSE of the recovered deflection at the three input locations, as shown in Figure 5.

The bars and error bars indicate the means and standard deviations, respectively. In the physical domain, *W* = 51 caused excessive smoothing. The PCC at each point decreased to approximately 0.91, while both the NRMSE and its dispersion increased markedly. With *W* = 15, the PCC exceeded 0.98 and the NRMSE remained below 2% at every point, showing that this configuration suppressed noise while preserving the main waveform features. In the simulation domain, numerical differentiation and recovery introduced an amplitude bias, as shown in Figure 4f, and the NRMSE exceeded 7%. Nevertheless, the PCC remained above 0.96 at all points, indicating that the chosen parameters preserved the principal waveform features of the reference deflection. The time–frequency characteristics and statistical results together show that the SG preprocessing procedure provides stable equivalent-acceleration inputs for the temporal module.

## 3. Model and Training

To address the coupling of spatiotemporal derivatives of different orders and the challenge of stress reconstruction, we developed DS-PINN. The network decomposes multi-location response reconstruction into latent-space temporal evolution and spatial-basis representation, thereby avoiding the joint evaluation of the temporal and spatial derivatives in Equation (1) within a single network. This section presents the DS-PINN architecture, simulation pretraining, cross-domain fine-tuning, and training configuration.

### 3.1. DS-PINN Architecture

**Figure 6:**
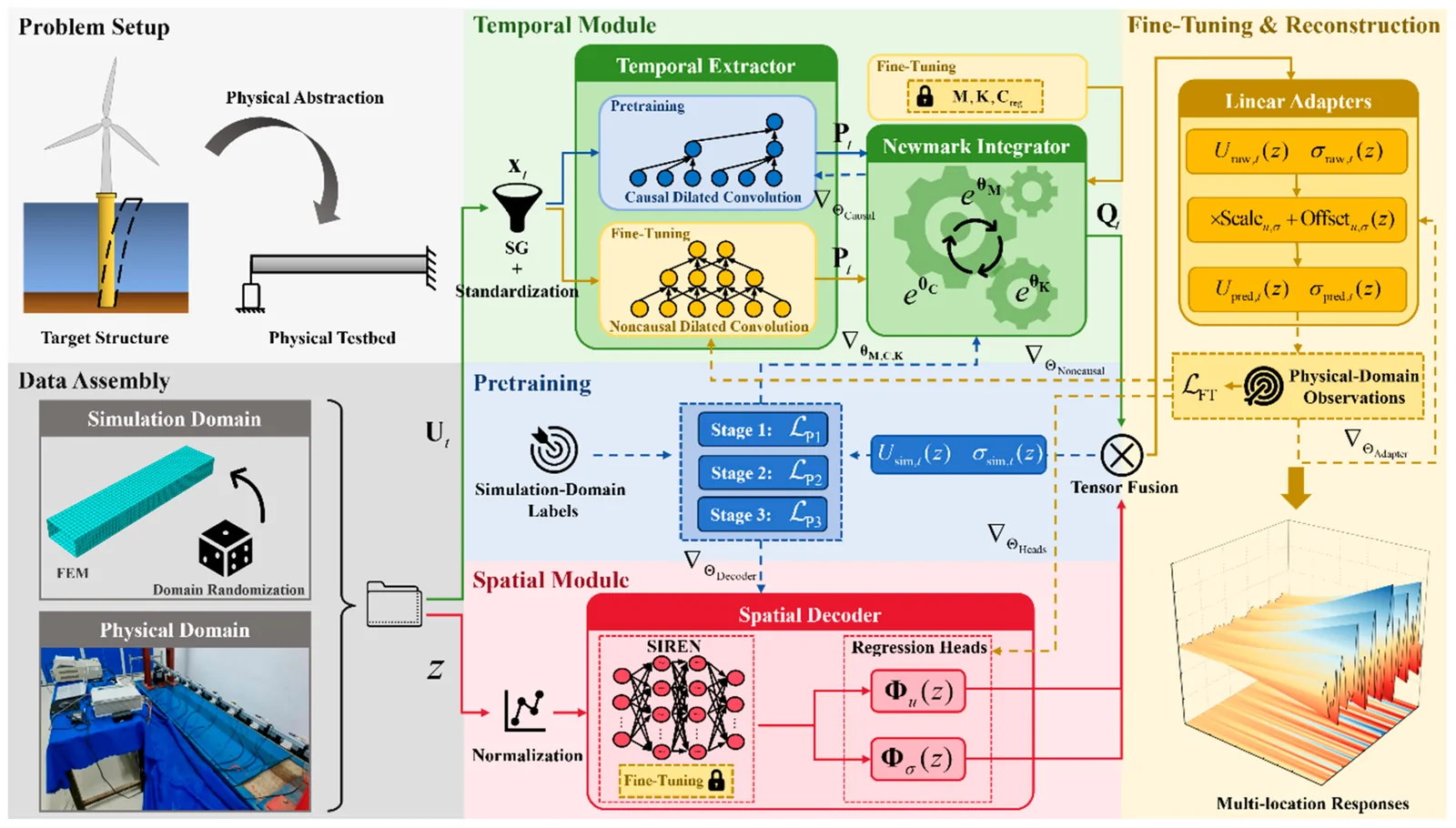
Framework of DS-PINN.

DS-PINN comprises a temporal module, a spatial module, and linear adapters, as shown in Figure 6. The temporal module receives sparse deflection histories from three measurement points. SG analytical differentiation and standardization transform these histories into the equivalent-acceleration sequence **x***_t_*. A temporal extractor maps this sequence to an *r*-dimensional generalized equivalent excitation **P***_t_*. A Newmark integration operator [31] then solves the latent-space dynamic equation to obtain time-evolving latent coordinates **Q***_t_*. In the spatial module, a decoder receives the continuous normalized coordinate *z*. A shared sinusoidal representation network (SIREN) [32] extracts spatial features, and two regression heads generate the deflection basis **Φ***_u_*(*z*) and stress basis **Φ***_σ_*(*z*). Tensor inner products between the latent coordinates and the two spatial bases yield deflection and stress at the specified locations. Linear adapters at the output further correct the amplitude and baseline of physical-domain responses.

DS-PINN uses Newmark integration to evolve second-order latent dynamics and separate spatial bases to reconstruct deflection and stress. This separation simplifies joint response learning compared with a single spatiotemporal coordinate network. Shared latent coordinates and spatial-basis consistency link the responses. During pretraining, a generalized latent energy-balance constraint also regularizes their temporal evolution. These elements introduce physical information into learning. DS-PINN is therefore a physics-guided latent reduced-order formulation that combines dual-response reconstruction with simulation-to-experiment adaptation; it does not directly enforce the Euler–Bernoulli PDE residual.

#### 3.1.1. Temporal Module

The temporal extractor is a data-driven feature-mapping network. It maps the standardized equivalent acceleration **x***_t_* to the *r*-dimensional generalized equivalent excitation **P***_t_*. The network does not directly predict temporal evolution, which is instead calculated by the subsequent Newmark integration operator.

During simulation pretraining, causal dilated convolution was applied. One-sided padding maintained temporal causality:

(7) $$P_{t}=f\left(\sum_{i=0}^{n_{k}-1}W_{c,i}^{\mathrm{causal}}x_{t-d\cdot i}+b_{c}^{\mathrm{causal}}\right)$$

During cross-domain fine-tuning and testing, the Newmark integration operator and SIREN backbone inherited and froze their pretrained weights. The temporal extractor was replaced by a noncausal dilated convolution. This convolution operated on the complete input sequence and used symmetric padding to integrate local preceding and subsequent information, accommodating temporal differences between the simulation and physical domains:

(8) $$P_{t}=f\left(\sum_{i=-(n_{k}-1)/2}^{(n_{k}-1)/2}W_{c,i}^{\mathrm{noncausal}}x_{t-d\cdot i}+b_{c}^{\mathrm{noncausal}}\right)$$

where *n_k_* is the convolution-kernel size, *d* is the dilation factor, and $W_{c,i}^{\mathrm{causal}}$, $b_{c}^{\mathrm{causal}}$, $W_{c,i}^{\mathrm{noncausal}}$, and $b_{c}^{\mathrm{noncausal}}$ are the convolution weights and biases for the two stages. *f*(⋅) is the Gaussian error linear unit (GELU) activation function.

To reduce error accumulation by a purely data-driven temporal network during long-sequence calculations, the temporal module incorporated a Newmark integration operator. Given **P***_t_*, this operator updated the latent coordinates through the second-order dynamic equation:

(9) $$M{\ddot{Q}}_{t}+C{\dot{Q}}_{t}+KQ_{t}=P_{t}$$

where **Q***_t_* contains the temporal coefficients of the learned deflection and stress spatial bases, and ${\dot{Q}}_{t}$ and ${\ddot{Q}}_{t}$ are their first and second time derivatives. For the elastic beam responses considered here, shared temporal coefficients describe how deflection and stress evolve. A second-order formulation draws on the inertia, damping, and restoring effects of structural dynamics, with **M**, **C**, and **K** representing the corresponding matrices in latent space. The coefficients and spatial bases are learned to reconstruct responses; a one-to-one correspondence with physical vibration modes has not been established. To keep the effective matrix **M**_eff_ positive definite, **M** was fixed as the identity matrix, while **C** and **K** were parameterized as positive diagonal matrices.

The integration operator used the Newmark-β average-acceleration method, with *γ* = 0.5 and *β* = 0.25. Prediction and correction over a discrete time step Δ*t* proceeded as follows.

First, the predicted latent coordinates and latent velocity were calculated:

(10) $${\tilde{Q}}_{t+\Delta t}=Q_{t}+\Delta t{\dot{Q}}_{t}+\frac{\Delta t^{2}}{2}(1-2\beta ){\ddot{Q}}_{t}$$

(11) $${\tilde{\dot{Q}}}_{t+\Delta t}={\dot{Q}}_{t}+\Delta t(1-\gamma ){\ddot{Q}}_{t}$$

Second, the effective matrix was calculated:

**M**_eff_ = **M** + *γ*Δ*t***C** + *β*Δ*t*^2^**K** (12)

Third, the latent acceleration at the next time step was solved. Because the matrix was diagonal, inversion reduced to scalar division:

(13) $${\ddot{Q}}_{t+\Delta t}=M_{\mathrm{eff}}^{-1}\left[P_{t+\Delta t}-C{\tilde{\dot{Q}}}_{t+\Delta t}-K{\tilde{Q}}_{t+\Delta t}\right]$$

Finally, a second correction was applied:

(14) $$Q_{t+\Delta t}={\tilde{Q}}_{t+\Delta t}+\beta \Delta t^{2}{\ddot{Q}}_{t+\Delta t}$$

(15) $${\dot{Q}}_{t+\Delta t}={\tilde{\dot{Q}}}_{t+\Delta t}+\gamma \Delta t{\ddot{Q}}_{t+\Delta t}$$

Through this recurrence, **P***_t_* was transformed into a continuously evolving latent-coordinate sequence **Q***_t_*, providing a stable temporal representation for long-sequence response reconstruction.

#### 3.1.2. Spatial Module and Tensor Fusion

The spatial module receives the normalized coordinate *z* ∈ [−1,1] and uses a SIREN to extract continuous spatial features:

**h**_1_(*z*) = sin(*ω*_0_**W**_s,1_*z* + **b**_s,1_) (16)

(17) $$\begin{array}{cc} h_{l}(z)=\sin(W_{s,l}h_{l-1}(z)+b_{s,l}) & l=2,\ldots ,D \end{array}$$

where **h***_l_*(*z*) is the output of hidden layer *l*, **W***_s_*_,*l*_ and **b***_s_*_,*l*_ are the corresponding weights and biases, *ω*_0_ is the frequency-scaling factor of the first layer, and *D* is the number of network layers.

The spatial decoder comprises a shared SIREN backbone and two regression heads, which output the *r*-dimensional deflection basis **Φ***_u_*(*z*) and stress basis **Φ***_σ_*(*z*). During the forward pass, stress is output directly by an independent regression head and does not require second-order automatic spatial differentiation of the deflection field.

Under the variable-separation assumption, tensor inner products between the latent coordinates and spatial bases yield the initial deflection field *U*_raw,*t*_(*z*) and stress field *σ*_raw,*t*_(*z*) at discrete time *t*:

(18) $$U_{\mathrm{raw},t}(z)=\sum_{i=1}^{r}Q_{i,t}\cdot \phi_{u,i}(z)$$

(19) $$\sigma_{\mathrm{raw},t}(z)=\sum_{i=1}^{r}Q_{i,t}\cdot \phi_{\sigma ,i}(z)$$

where *Q_i_*_,*t*_ is component *i* of **Q***_t_*, and *ϕ_u_*_,*i*_(*z*) and *ϕ_σ_*_,*i*_(*z*) are the *i*th deflection and stress basis functions. The latent dimension *r* determines the representation capacity, with *r* temporal coefficients serving as weights for the corresponding deflection and stress spatial basis functions. The spatial decoder provides a continuous coordinate-based representation. Fine-tuning and testing used different experimental records at the same 11 evaluation locations, and inference required only three deflection channels. The experimental validation therefore assesses reconstruction across records at these 11 locations.

#### 3.1.3. Linear Adapters

Linear adapters were applied separately to deflection and stress at the network output to correct differences in response magnitude and static baseline between the simulation and physical domains. At the 11 evaluation locations in the physical domain, learnable global scale factors Scale*_u_* and Scale*_σ_*, together with position-dependent offsets Offset*_u_*(*z*) and Offset*_σ_*(*z*), linearly transformed the initial reconstruction:

*U*_pred,*t*_(*z*) = *U*_raw,*t*_(*z*) ⋅ Scale*_u_* + Offset*_u_*(*z*) (20)

*σ*_pred,*t*_(*z*) = *σ*_raw,*t*_(*z*) ⋅ Scale*_σ_* + Offset*_σ_*(*z*) (21)

where *U*_pred,*t*_(*z*) and *σ*_pred,*t*_(*z*) are the final reconstructed responses. The linear adapters correct amplitude and baseline biases arising from differences in measurement conditions and response distributions between the two domains, improving adaptation to physical-domain responses.

### 3.2. Simulation Pretraining

Pretraining used 2000 simulated 60 s records, of which 1800 were used for training and 200 for validation. A three-stage strategy progressively introduced deflection supervision, stress supervision, a spatial soft constraint, and an energy constraint. The mean-squared error (MSE) quantified the discrepancy between a reconstructed sequence and its ground-truth labels:

(22) $$\mathrm{MSE}(y_{\mathrm{pred}},y_{\mathrm{gt}})=\frac{1}{N}\sum_{i=1}^{N}{\left(y_{\mathrm{pred},i}-y_{\mathrm{gt},i}\right)}^{2}$$

where *N* is the total number of samples.

Stage 1: The matrices **M**, **K**, and **C** in the Newmark operator were initialized and frozen. Only the temporal extractor and spatial decoder were trained using deflection supervision, allowing the network to first establish a basic response mapping. The overall loss was the deflection loss:

ℒ_P1_ = MSE(*U*_raw,*t*_(*z*),*U*_sim,*t*_(*z*)) (23)

where *U*_raw,*t*_(*z*) is the initial reconstructed deflection from Equation (18), and *U*_sim,*t*_(*z*) is the simulated deflection label.

Stage 2: To coordinate relative changes in the deflection-curvature basis and stress basis, the parameters available in Stage 1 remained trainable. A learnable scaling coefficient *η* was introduced, and the concurrently generated bases **Φ***_u_*(*z*) and **Φ***_σ_*(*z*) defined the spatial-basis consistency loss:

(24) $$L_{\mathrm{spa}}=\mathrm{MSE}\left(\Phi_{\sigma }(z),\eta \frac{\partial^{2}\Phi_{u}(z)}{\partial z^{2}}\right)$$

Stress supervision, ℒ*_σ_* = MSE(*σ*_raw,*t*_(*z*),*σ*_sim,*t*_(*z*)), was also introduced. To reduce the discrepancy between the gradient magnitudes of the deflection and stress losses, adaptive weights *w_u_* and *w_σ_* were dynamically adjusted using GradNorm [33], with a smoothing coefficient of 0.12. The Stage 2 loss was:

ℒ_P2_ = *w_u_*ℒ_P1_ + *w_σ_*ℒ*_σ_* + *λ*_spa_ℒ_spa_ (25)

where *σ*_raw,*t*_(*z*) is the initial reconstructed stress from Equation (19), *σ*_sim,*t*_(*z*) is the simulated stress label, and *λ*_spa_ is the spatial soft-constraint weight.

Stage 3: The matrices **K** and **C** in the Newmark integrator were unfrozen. A generalized latent-space energy-balance residual derived from Equation (9) was used to regularize the joint updates of the generalized matrices and latent coordinates. The generalized latent-space energy at discrete time *t* was defined as:

(26) $$E_{g,t}=\frac{1}{2}{{\dot{Q}}_{t}}^{Τ}M{\dot{Q}}_{t}+\frac{1}{2}{Q_{t}}^{Τ}KQ_{t}$$

The corresponding generalized input and dissipation powers in latent space were defined as:

(27) $$P_{\mathrm{in},t}={{\dot{Q}}_{t}}^{Τ}P_{t}$$

(28) $$P_{\mathrm{diss},t}={{\dot{Q}}_{t}}^{Τ}C{\dot{Q}}_{t}$$

The balance among the rate of change of the generalized latent-space energy, input power, and dissipation power defined the generalized energy loss:

(29) $$L_{\mathrm{energy}}=E_{t}\left[{\left(\frac{\frac{E_{g,t}-E_{g,t-\Delta t}}{\Delta t}-(P_{\mathrm{in},t}-P_{\mathrm{diss},t})}{{\bar{E}}_{g}+\varepsilon }\right)}^{2}\right]$$

The energy constraint regularizes latent-state evolution by penalizing the difference between the rate of change of latent energy and the input power remaining after damping dissipation.

The Stage 3 loss was:

ℒ_P3_ = ℒ_P2_ + *λ*_energy_ℒ_energy_ (30)

where E*_t_* denotes averaging over time, Δ*t* is the integration step, ${\bar{E}}_{g}$ is the mean generalized energy over the current interval, *ε* = 10^−6^ prevents division by zero, and *λ*_energy_ is the generalized energy-loss weight. These quantities are auxiliary constructs in latent space and are used only for numerical regularization; they are not interpreted as the physical mass, damping, stiffness, or energy of the beam.

### 3.3. Cross-Domain Fine-Tuning

The physical-domain dataset comprised 25 measured 60 s records. To remove electrical surge interference during startup, the first 5 s of each record were discarded, leaving a valid duration of 55 s. Four complete records were reserved as an independent test set. The remaining 21 records were divided into 16 fine-tuning training records and 5 validation records.

To reduce computational cost, only a 16 s interval containing the principal response was retained from each training and validation record. The baseline offset of the midspan deflection was first removed. The excitation onset was defined as the first time at which its absolute value reached 5% of the absolute temporal peak. All three input channels and all response channels were then extracted from 1 s before to 15 s after this onset. The complete 55 s records were retained in the independent test set to evaluate long-sequence response reconstruction.

During fine-tuning, only the temporal extractor, linear adapters, the scaling coefficient *η* in Equation (24), and the two regression heads of the spatial decoder were trainable. In the Newmark operator, **M** = **I** and **K** remained frozen. To reduce the accumulation of high-frequency physical-domain disturbances during latent-coordinate recurrence, **C** was replaced by the fixed diagonal regularization matrix:

(31) $$C_{\mathrm{reg}}=\left(\frac{2}{r}\sum_{i=1}^{r}\sqrt{k_{i}m_{i}}\right)I_{r\times r}$$

where *k_i_* and *m_i_* are the *i*th diagonal elements of **K** and **M**, respectively, and **I***_r_*_×*r*_ is the identity matrix. This configuration applies uniform numerical attenuation to each latent dimension, reducing the effect of high-frequency disturbances on long-sequence reconstruction.

During fine-tuning, the temporal extractor adapts to differences in temporal responses, while the linear adapters correct differences in magnitude and baseline. Varying the simulated density and root rotational stiffness broadens the pretraining distribution. Numerical damping helps suppress high-frequency disturbances during latent-state evolution. The fine-tuning loss ℒ_FT_ comprised four terms:

(32) $$L_{\mathrm{VMSE}}=\frac{\mathrm{MSE}(U_{\mathrm{pred},t}(z),U_{\mathrm{gt},t}(z))}{\mathrm{Var}(U_{\mathrm{gt},t}(z))+\varepsilon }+\frac{\mathrm{MSE}(\sigma_{\mathrm{pred},t}(z),\sigma_{\mathrm{gt},t}(z))}{\mathrm{Var}(\sigma_{\mathrm{gt},t}(z))+\varepsilon }$$

ℒ_PCC_ = (1 − PCC(*U*_pred,*t*_(*z*),*U*_gt,*t*_(*z*))) + (1 − PCC(*σ*_pred,*t*_(*z*),*σ*_gt,*t*_(*z*))) (33)

(34) $$L_{\mathrm{Bias}}=\mathrm{MSE}({\bar{U}}_{\mathrm{pred}}(z),{\bar{U}}_{\mathrm{gt}}(z))+\mathrm{MSE}({\bar{\sigma }}_{\mathrm{pred}}(z),{\bar{\sigma }}_{\mathrm{gt}}(z))$$

ℒ_FT_ = ℒ_VMSE_ + ℒ_PCC_ + ℒ_Bias_ + *λ*_spa_ℒ_spa_ (35)

where *U*_pred,*t*_(*z*) and *σ*_pred,*t*_(*z*) are the reconstructed responses from Equations (20) and (21), and *U*_gt,*t*_(*z*) and *σ*_gt,*t*_(*z*) are the measured labels. An overbar denotes the temporal mean. ℒ_VMSE_ balances the different scales of deflection and stress, ℒ_PCC_ constrains waveform correlation, ℒ_Bias_ corrects temporal-mean deviations at each measurement point, and ℒ_spa_ limits relative changes between the two spatial bases.

### 3.4. Network and Training Configuration

Model training and testing were performed on a computing node equipped with an NVIDIA A100-SXM4 GPU with 40 GB of memory and an AMD EPYC 7K62 CPU. The implementation used PyTorch 2.4 with CUDA 12.1 acceleration.

The latent-space dimension was *r* = 8. During pretraining, the causal temporal extractor contained three one-dimensional dilated-convolution layers. The kernel size was 3, the number of hidden channels was 16, and the dilation factors were 1, 2, and 4. During fine-tuning, the noncausal extractor contained 6 dilated-convolution layers with a kernel size of 3. The first layer had 16 hidden channels, the remaining layers had 32, and the dilation factors were 1, 2, 4, 8, 16, and 32. The spatial decoder used a SIREN backbone with two regression heads. The backbone comprised 4 fully connected layers with a hidden dimension of 64, and the first-layer frequency-scaling factor was *ω*_0_ = 30.0.

AdamW was used throughout training. The three pretraining stages contained 50, 50, and 100 epochs, respectively, with a batch size of 128. Learning rates were isolated by stage and module. The initial learning rate was 1 × 10^−3^ in Stage 1 and 5 × 10^−4^ in Stage 2. In Stage 3, the temporal extractor, spatial decoder, and scaling coefficient *η* in Equation (24) used a learning rate of 1 × 10^−4^, while the Newmark integrator used 5 × 10^−5^. The spatial constraint weight was *λ*_spa_ = 0.5 in Stages 2 and 3, and the energy-loss weight was *λ*_energy_ = 0.01 in Stage 3.

For cross-domain fine-tuning, the model inherited the final pretrained weights and applied the module replacements and parameter settings described in Section 3.3. Fine-tuning comprised 250 epochs with a batch size of 5. The learning rate was 2 × 10^−3^ for the temporal extractor and 1 × 10^−4^ for the two spatial-decoder regression heads and *η*. Within the linear adapters, the learning rates for Scale and Offset(*z*) were 0.5 and 0.1, respectively. The weight *λ*_spa_ remained 0.5. After every epoch, the sum of the deflection and stress PCC values was evaluated on the 5 validation records. The model with the highest value of this criterion was retained as the final model.

## 4. Results and Discussion

The multi-location reconstruction performance of DS-PINN was evaluated using measured data. The evaluation comprised hyperparameter sensitivity analysis, verification of the training strategy, long-duration reconstruction, ablation of core components, and comparisons with benchmark models. The PCC, NRMSE, PeakErr, and FreqErr were calculated as defined in Section 2.3 and averaged over the 4 independent test records. Residual distributions, regression scatter plots, and spatial NRMSE values were calculated from the pooled results. Spatial NRMSE was normalized separately by the maximum absolute reference response across all pooled test samples and the 11 evaluation locations.

### 4.1. Hyperparameter Sensitivity and Training-Strategy Verification

**Figure 7:**
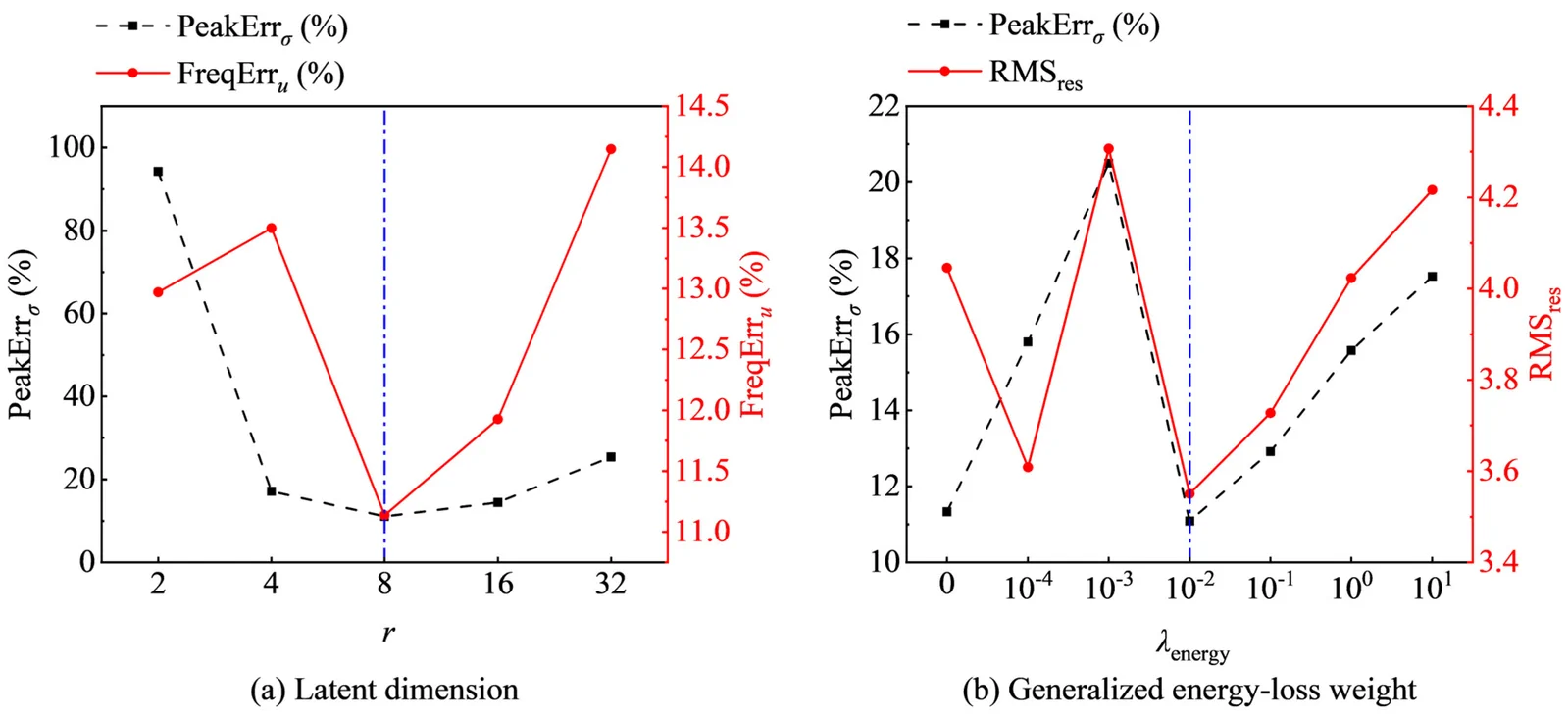
Post hoc sensitivity analysis of DS-PINN hyperparameters.

Figure 7 presents a post hoc sensitivity analysis of the latent-space dimension *r* and the generalized energy-loss weight *λ*_energy_. The nominal configuration, *r* = 8 and *λ*_energy_ = 0.01, was specified before test-set evaluation. Each alternative configuration underwent independent pretraining and fine-tuning before being evaluated on the same 4 test records. These additional runs were used only to assess sensitivity to the two hyperparameters and did not inform this configuration.

As shown in Figure 7a, the latent-space representation had insufficient capacity at *r* = 2, resulting in a stress PeakErr of 94.29%. At *r* = 8, the stress PeakErr and deflection FreqErr reached their lowest observed values of 11.09% and 11.14%, respectively. Both metrics increased as the dimension was further enlarged. At *r* = 32, the stress PeakErr rose to 25.40%, indicating that increasing the latent dimension beyond *r* = 8 did not improve test performance.

With the latent dimension fixed at *r* = 8, we examined the sensitivity to *λ*_energy_. According to Equation (29), the root-mean-square generalized latent-space energy-balance residual was defined as:

(36) $${\mathrm{RMS}}_{\mathrm{res}}=\sqrt{L_{\mathrm{energy}}}$$

This metric represents the overall magnitude of the generalized latent-space energy-balance residual. As shown in Figure 7b, the stress PeakErr and RMS_res_ varied nonmonotonically with the loss weight. At *λ*_energy_ = 0.001, the stress PeakErr increased to 20.50%. Both metrics reached their lowest observed values at *λ*_energy_ = 0.01 and increased again at higher weights. These results therefore indicate that the prespecified nominal configuration provided a favourable balance between reconstruction error and the generalized latent-space energy-balance residual.

**Figure 8:**
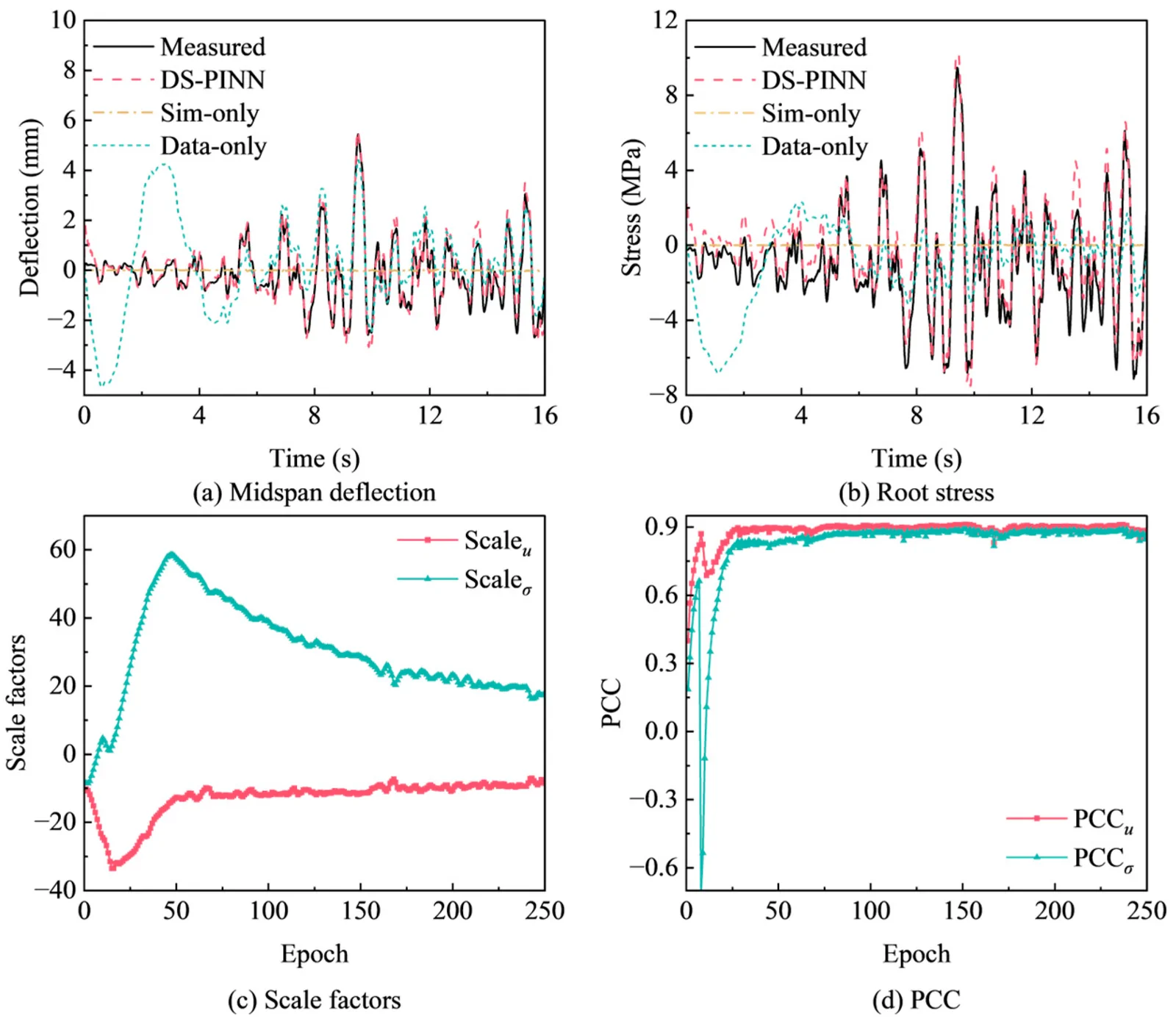
Comparison of training strategies and fine-tuning convergence.

Using this configuration, three models trained under different strategies were then evaluated for physical-domain response reconstruction. A representative 16 s measured segment was selected from the fine-tuning set to illustrate the reconstruction of midspan deflection and root stress. Sim-only denotes the pretrained model without physical-domain fine-tuning. Data-only denotes a DS-PINN backbone trained end-to-end on the physical-domain data without simulation pretraining. DS-PINN used the complete strategy of simulation pretraining followed by physical-domain fine-tuning. As shown in Figure 8a,b, Sim-only substantially underestimated the response amplitude, indicating that the pretrained model could not directly adapt to physical-domain responses without fine-tuning. Data-only exhibited low-frequency distortion and amplitude attenuation, indicating that the available measured dataset was insufficient to establish a stable multi-location response mapping from physical-domain training alone. DS-PINN, in contrast, closely tracked the principal waveforms of measured deflection and stress. Combining simulation pretraining with physical-domain fine-tuning therefore improved response reconstruction under sparse spatial measurements and limited measured data.

As shown in Figure 8c,d, the scale factors adjusted rapidly during the first 50 epochs and then stabilized. The validation PCC values for deflection and stress increased to approximately 0.9 and remained stable, indicating that fine-tuning had essentially converged.

### 4.2. Long-Duration Response Reconstruction

**Figure 9:**
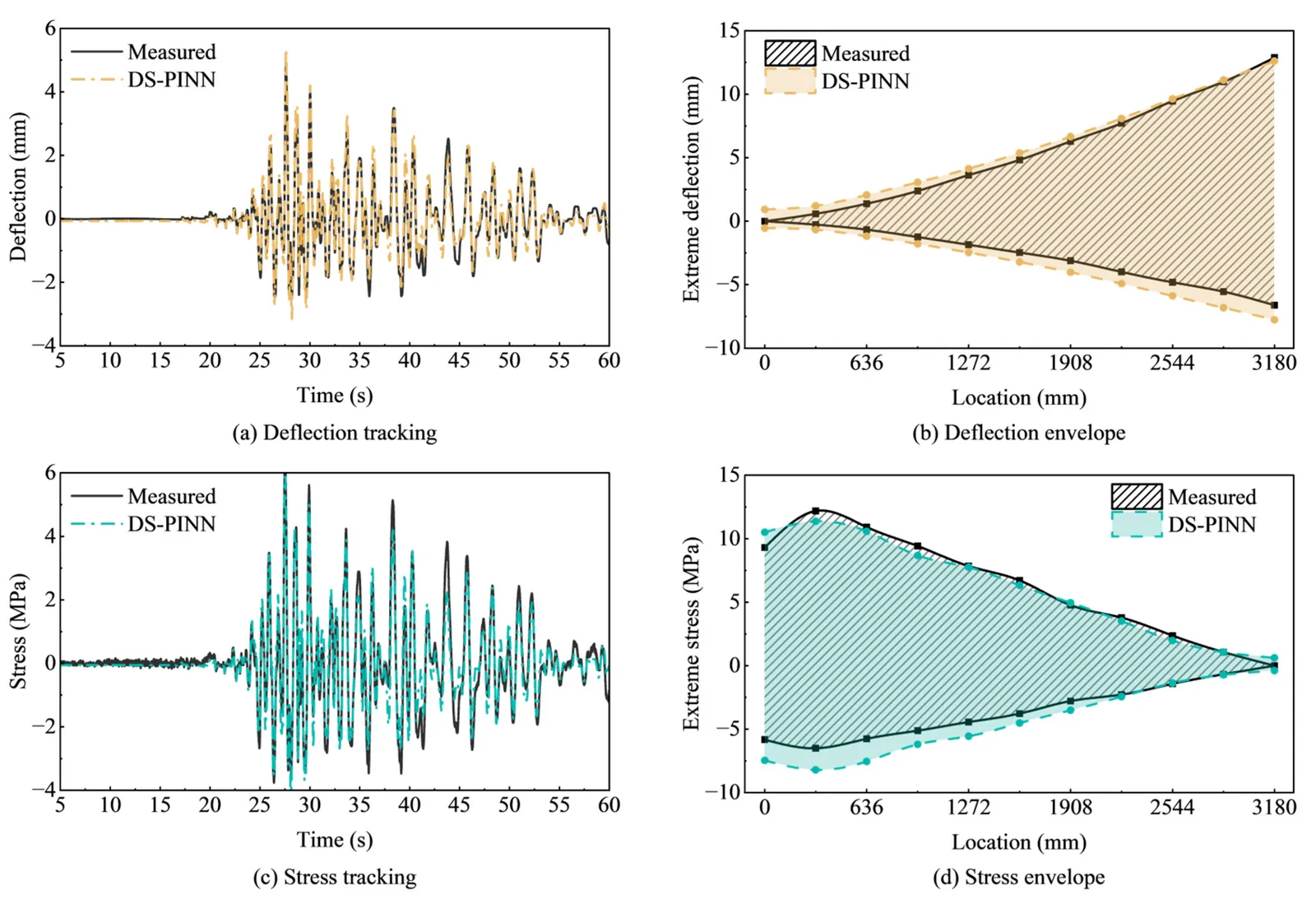
Long-duration response reconstruction and spatial envelopes.

A representative record from the four independent test records was selected for multi-location reconstruction over its valid 55 s duration. As shown in Figure 9a,c, the reconstructed deflection and stress generally followed the principal waveforms and amplitude variations of the measured responses. No marked cumulative phase drift or amplitude divergence occurred over the long record. The reconstructed and measured envelopes also showed consistent spatial trends, as shown in Figure 9b,d. In addition, the measured peak-stress location shifted inward from the root, and the model reproduced this spatial feature.

**Figure 10:**
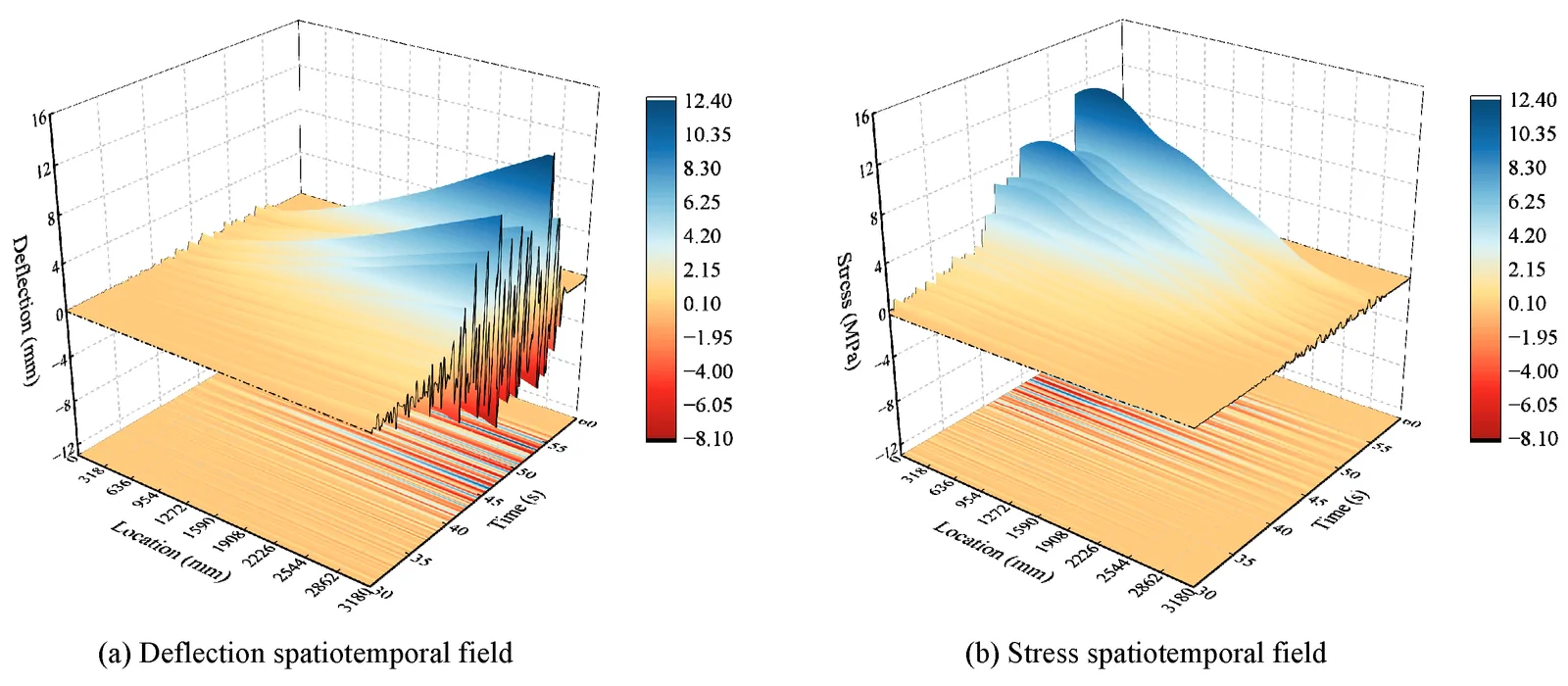
Reconstructed spatiotemporal response fields.

Figure 10 presents a second test record. Predictions at the 11 evaluation locations were interpolated to show how deflection and stress vary over time and along the beam.

**Figure 11:**
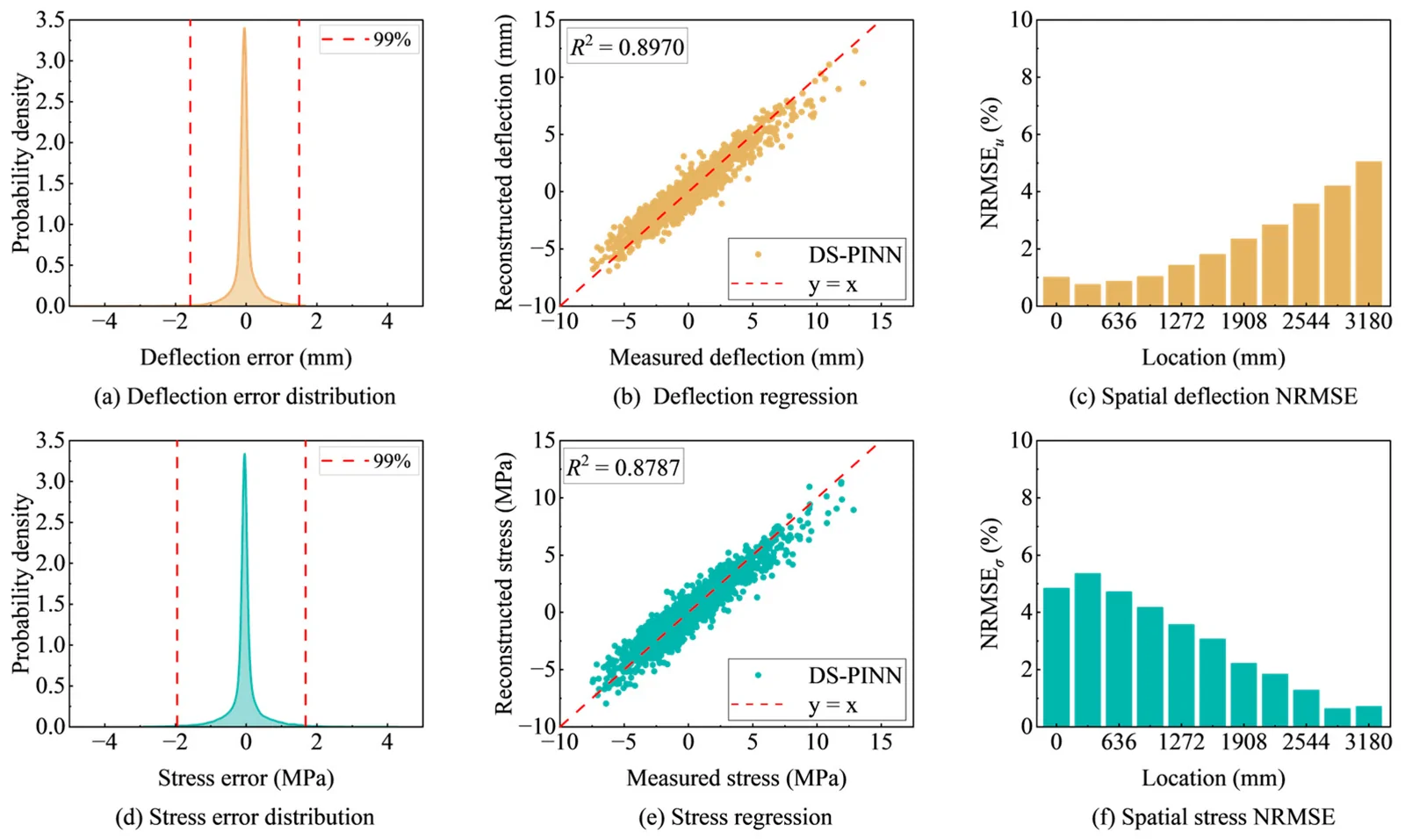
Global statistical evaluation of reconstruction errors.

The 4 independent test records were then pooled for a statistical evaluation of overall reconstruction error. As shown in Figure 11a,d, the probability densities of deflection and stress residuals were concentrated near zero. The red dashed lines denote the corresponding empirical 99% quantile intervals, and the overall residuals showed no pronounced systematic bias. In Figure 11b,e, the reconstructed and measured values were distributed mainly around *y* = *x*, with coefficients of determination of *R*^2^ = 0.8970 for deflection and *R*^2^ = 0.8787 for stress. Figure 11c,f show that the spatial NRMSE values of deflection and stress remained below 6% at all 11 measurement locations. These residual and regression results indicate that the model reconstructed deflection and stress at multiple locations from only three deflection input channels.

### 4.3. Ablation Analysis of Core Components

Table 2 lists the configurations of A1–A4 and the complete model, DS. A1 changes the temporal module, spatial-network width, and pretraining constraints. A2–A4 assess spatial-basis consistency, causal temporal extraction, and linear-adapter settings, respectively. Each configuration underwent independent pretraining and fine-tuning.

Each configuration used 1800 simulated records for training and 200 for validation, followed by fine-tuning with 16 experimental training records and 5 validation records. Loss functions and optimization settings followed Section 3.2, Section 3.3 and Section 3.4, with the differences specified in Table 2. For DS, fine-tuning updated the temporal extractor, both spatial regression heads, the spatial-consistency scaling coefficient, and the linear adapters. A1 also updated the LSTM at a learning rate of 0.0001. A2 excluded consistency-related optimization. A3 used a causal extractor and updated the same components as DS, whereas A4 kept the linear adapters fixed.

**Figure 12:**
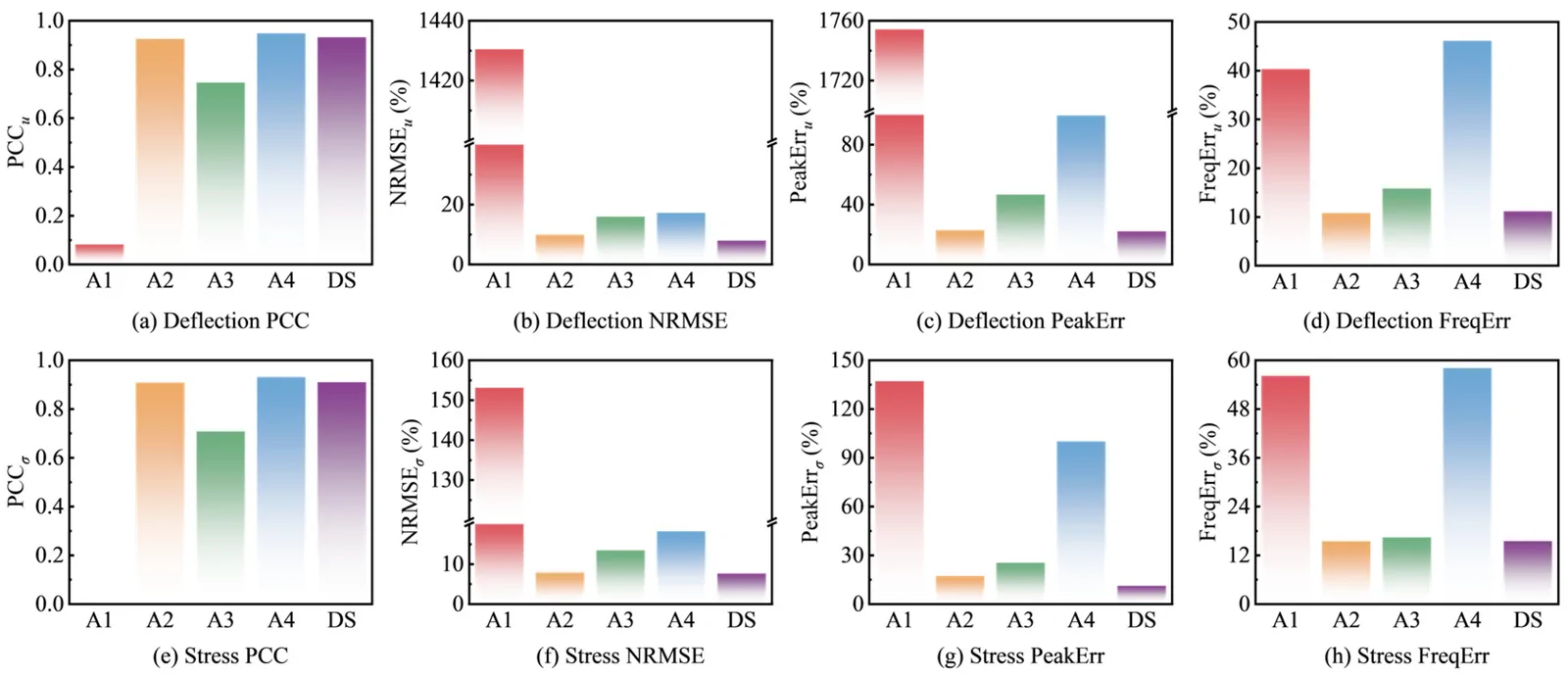
Comparison of reconstruction metrics across ablation variants.

Figure 12 compares the overall metrics of the five models. DS achieved PCC values of 0.9323 and 0.9093 for deflection and stress, respectively, with corresponding NRMSE values of 7.94% and 7.59%. It therefore provided a favourable balance between waveform correlation and overall error. When the Newmark integration operator was replaced by an LSTM, both PCC values for A1 approached zero and the NRMSE increased substantially, indicating a marked degradation in the temporal evolution of the latent coordinates. The PCC values of A3 decreased, and both NRMSE values exceeded 13%. In the present task, removing noncausal context during fine-tuning reduced the model’s ability to adapt to the temporal characteristics of the measured signals. The overall metrics of A2 were close to those of DS. Although A4 produced slightly higher PCC values, its deflection and stress NRMSE values increased to 17.22% and 18.18%, respectively, and both PeakErr and FreqErr increased markedly. A4 thus showed a clear separation between waveform correlation and amplitude accuracy.

**Figure 13:**
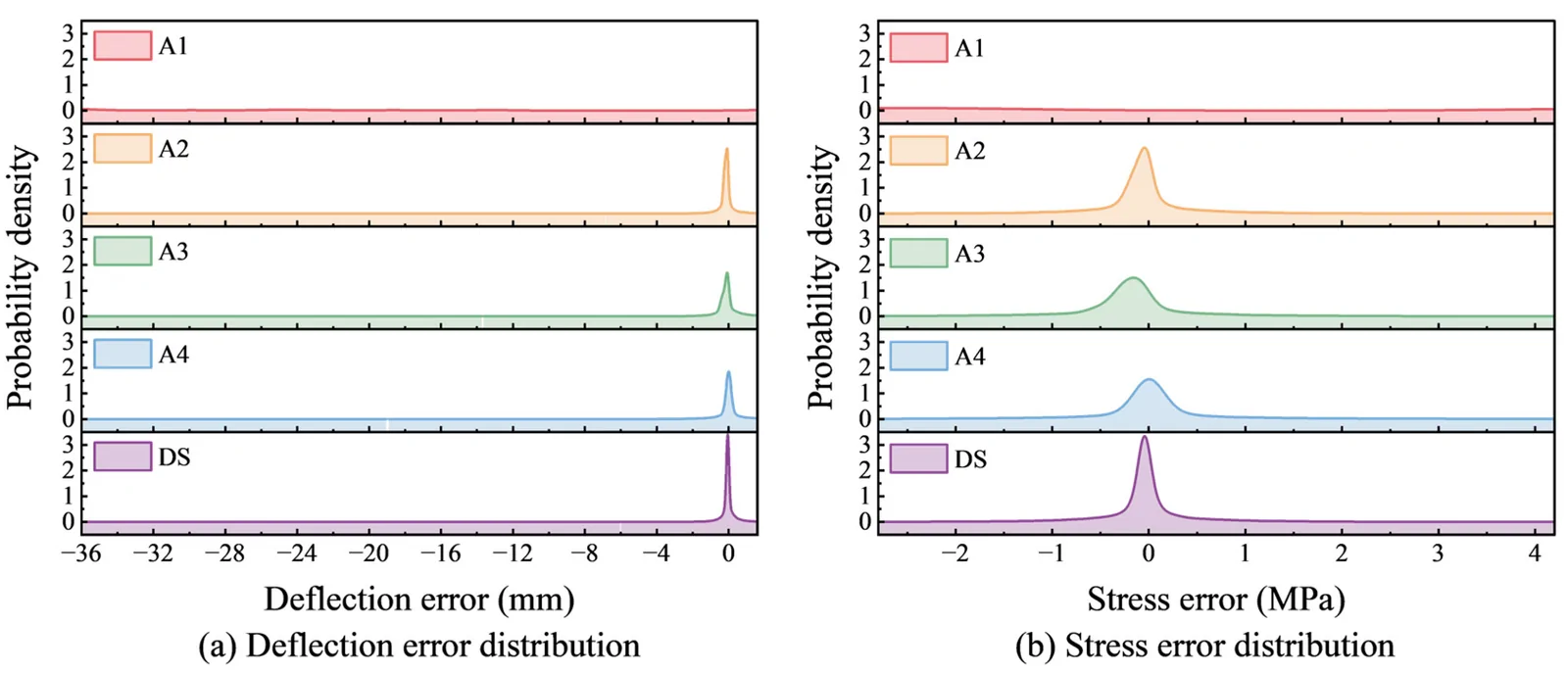
Residual probability density distributions of ablation variants.

Figure 13 shows the residual probability density distributions. The deflection and stress residuals of DS and A2 formed relatively narrow central peaks around zero. The A1 residuals were dispersed over a much wider range, while the distributions of A3 and A4 were noticeably broader than those of DS. Although residual distributions reflect the overall error range and dispersion, the distributions of A3 and A4 were similar. Their differences therefore required examination of the regression relationships.

**Figure 14:**
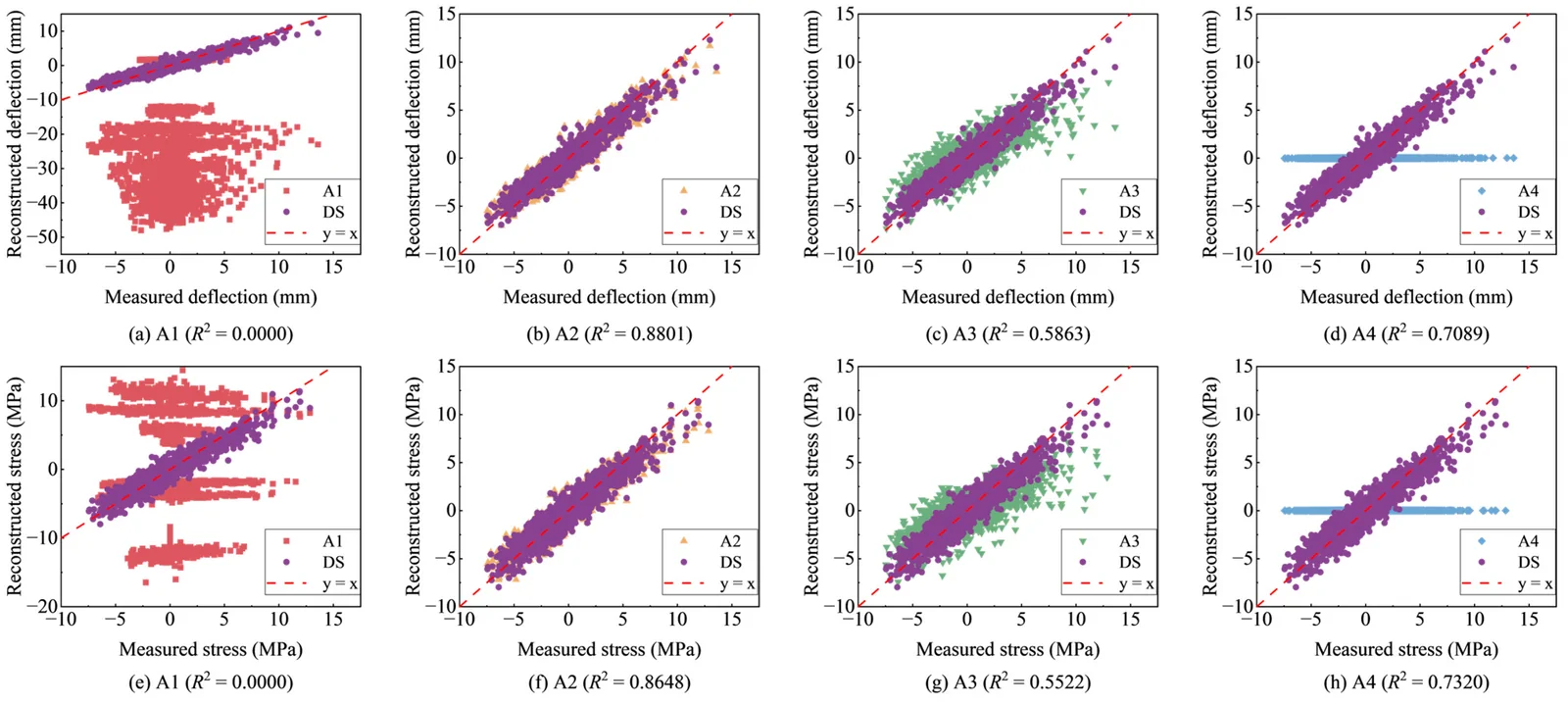
Regression comparison between ablation variants and DS-PINN.

As shown in Figure 14, the A1 scatter points deviated widely from *y* = *x*, and *R*^2^ approached zero, indicating that the stable mapping between reconstructed and measured values had been lost. The A2 points remained concentrated mainly along *y* = *x* and were similar to those of DS. The point cloud of A3 was substantially broader and deviated at larger response magnitudes, showing weaker temporal adaptation. A4 produced horizontal bands near a reconstructed value of zero, indicating substantial attenuation of some response amplitudes when the linear adapters were fixed during fine-tuning. Thus, A3 mainly increased the dispersion of reconstructed results, whereas A4 mainly lacked magnitude correction.

**Figure 15:**
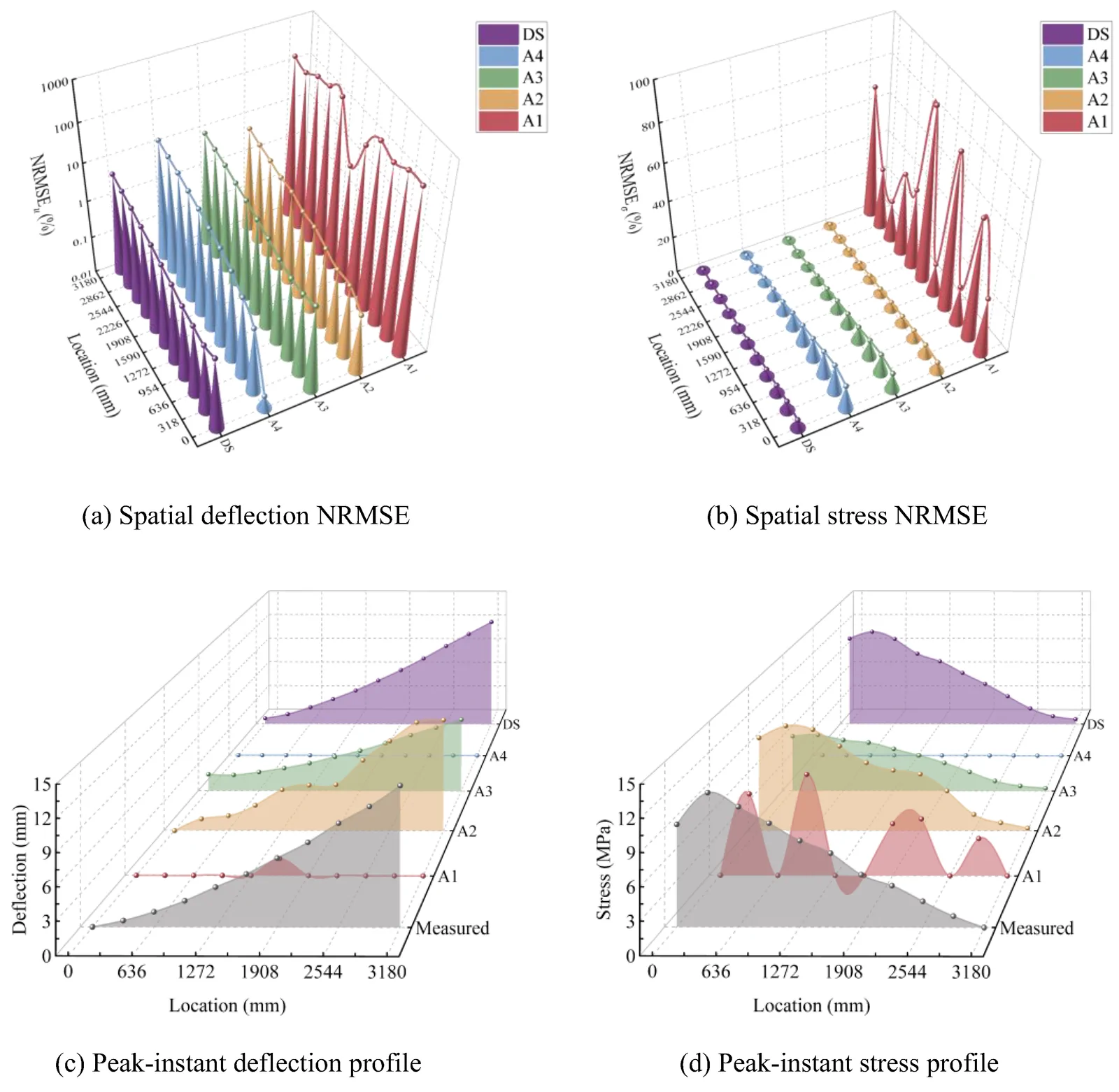
Spatial NRMSE distributions and response profiles at peak instants for ablation variants.

Figure 15 shows the spatial NRMSE and response profiles at peak instants. A1, A3, and A4 had larger spatial errors than DS at most locations, whereas A2 and DS were similar. Their spatial NRMSE values for both deflection and stress remained below 6%. At peak instants, however, the deflection and stress profiles of A2 exhibited local oscillations. DS maintained smoother spatial variations consistent with the measured trends. A3 retained the basic spatial pattern but showed larger local deviations. A4 produced substantial amplitude compression, while A1 exhibited major shape errors and spatial fluctuations. Although the full-duration spatial NRMSE values of A2 and DS were similar, their peak-instant profiles indicate that the spatial-basis consistency constraint reduced local spatial oscillations.

The Newmark integration operator governed temporal evolution, the noncausal extractor supported temporal adaptation, the linear adapters corrected response magnitude, and the spatial-basis constraint coordinated the spatial fields. Although DS-PINN did not rank first on every metric, its performance was more consistent across the evaluation criteria.

### 4.4. Comparison with Benchmark Models

DS-PINN was compared with the four benchmark models listed in Table 3. C1 and C2 used SG-derived equivalent accelerations from three deflection histories to predict deflection and stress at 11 evaluation locations. C3 took spatial and temporal coordinates as inputs and combined observations from one experimental record with PDE residual constraints. For C4, PSO calibrated an analytical beam model whose parameters were then held fixed during testing. All models were evaluated on the same test records using the same metrics.

C1 and C2 were trained with AdamW for 300 epochs using a batch size of 5. The initial learning rate was 0.001 and was halved at epochs 100 and 200. The loss combined variance-normalized MSE and correlation terms. Checkpoints were selected using the sum of the validation PCC values for deflection and stress. C3 was trained with AdamW for 300 updates at a learning rate of 0.001. Each update sampled 2000 observation points and 5000 collocation points, with a PDE-loss weight of 10^−4^. C4 used 150 PSO particles over 150 iterations to minimize the combined deflection and stress MSE. Inertia decreased from 0.9 to 0.4, and the cognitive and social coefficients were both 2.

**Figure 16:**
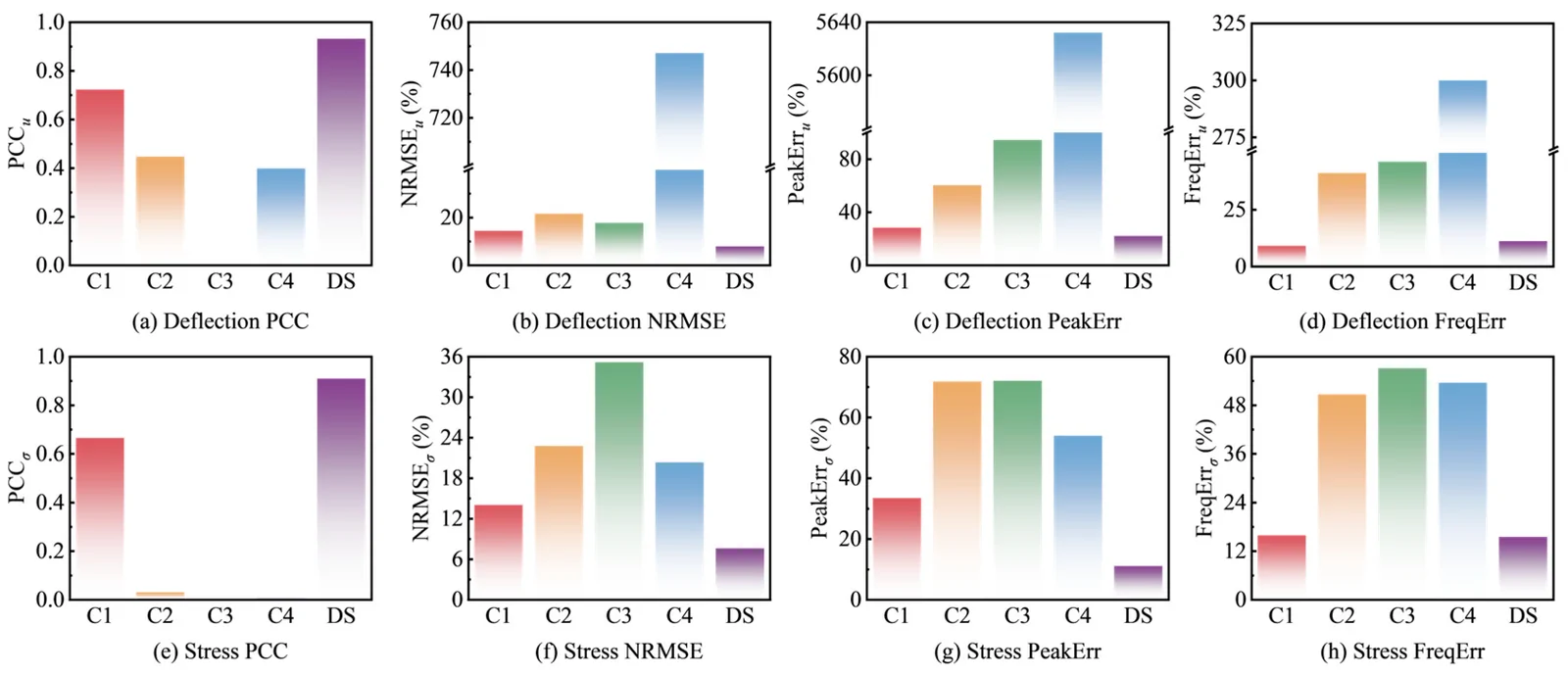
Comparison of reconstruction metrics across benchmark models.

Figure 16 compares the overall metrics of the five models. Across the 4 test records, the PCC values for DS-PINN were 0.9323 ± 0.0416 for deflection and 0.9093 ± 0.0596 for stress. The corresponding NRMSE values were 7.94% ± 2.04% and 7.59% ± 2.34%, respectively. These values are means ± sample standard deviations across records. C1 was the closest benchmark, with PCC values of 0.7231 and 0.6650 and NRMSE values of 14.44% and 14.03% for deflection and stress, respectively. Although the deflection FreqErr of C1 was slightly lower than that of DS, and its stress FreqErr was similar, the two PeakErr values increased to 28.31% and 33.41%. C1 therefore retained some dominant-frequency content but showed substantial errors in time-domain extrema. The stress PCC of C2 decreased to 0.0298, while both PCC values of C3 approached zero. C4 had a deflection NRMSE of 747.13% and a stress PCC of only 0.0156. The poor performance of C3 and C4 needs further verification. These results do not establish that DS-PINN outperforms PINN or PSO-based methods in general.

**Figure 17:**
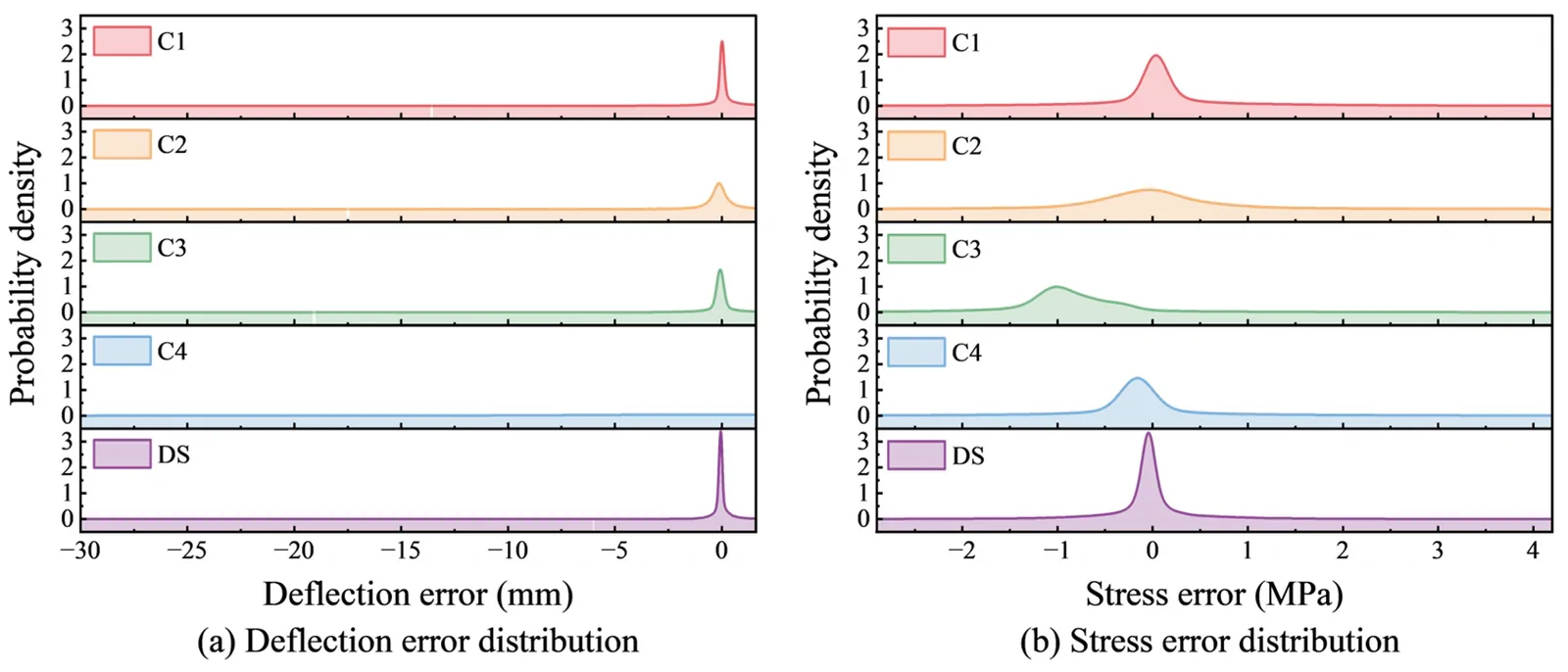
Residual probability density distributions of benchmark models.

**Figure 18:**
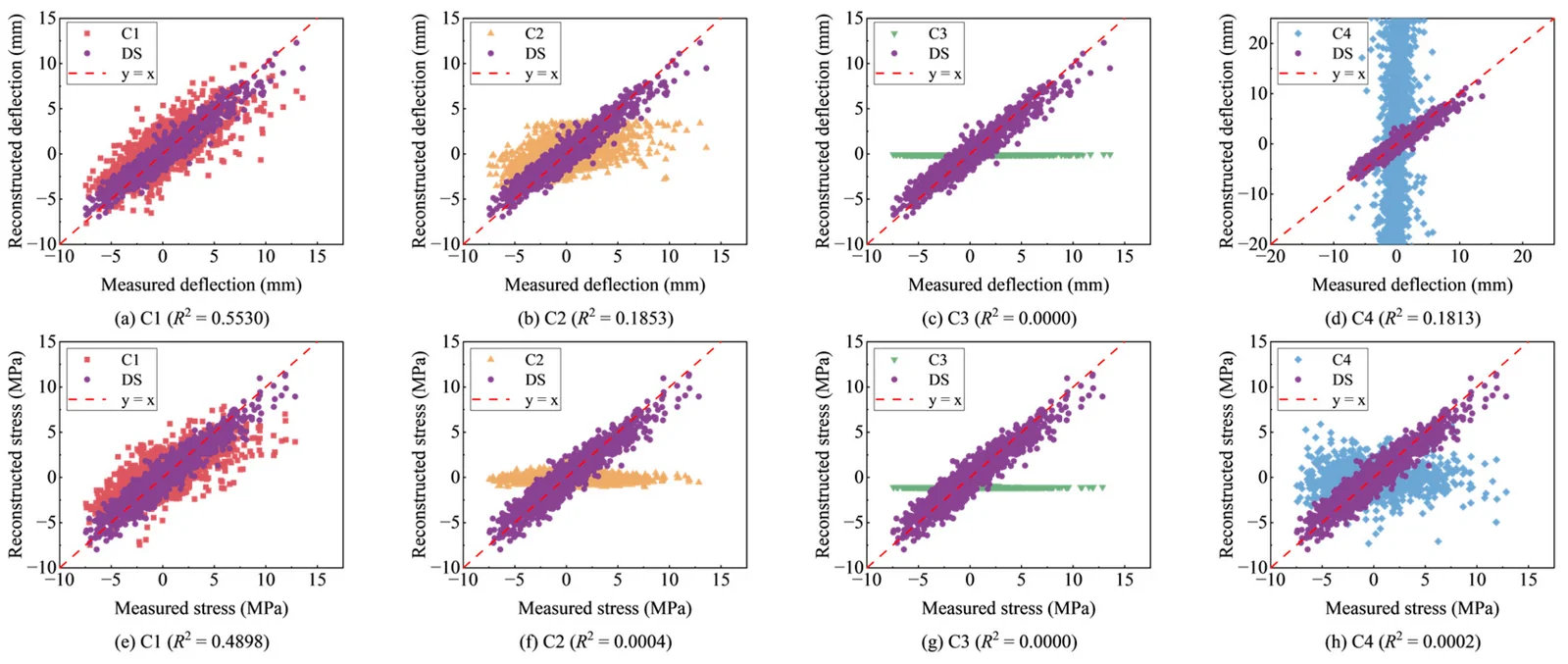
Regression comparison between benchmark models and DS-PINN.

Figure 17 further shows the residual probability density distributions. The deflection and stress residuals of DS formed the highest and narrowest central peaks around zero, followed by C1. The C2 distributions were substantially broader. The stress residuals of C3 shifted towards negative values, while the range of deflection errors for C4 expanded markedly. Together with Figure 18, these results show that the local peak near zero in the C3 deflection residuals arose mainly from low-amplitude reconstructions and did not indicate a correct response mapping.

The regression scatter plots in Figure 18 show that the C1 points remained distributed along *y* = *x*, but with substantially greater dispersion than DS. C1 therefore retained some response variation but had large errors across different amplitude ranges. C2 showed only a weak diagonal relationship for deflection, while its reconstructed stress tended towards low-amplitude output. Both C3 reconstructions were concentrated near zero and failed to track the measured responses. C4 likewise failed to produce a stable diagonal distribution.

**Figure 19:**
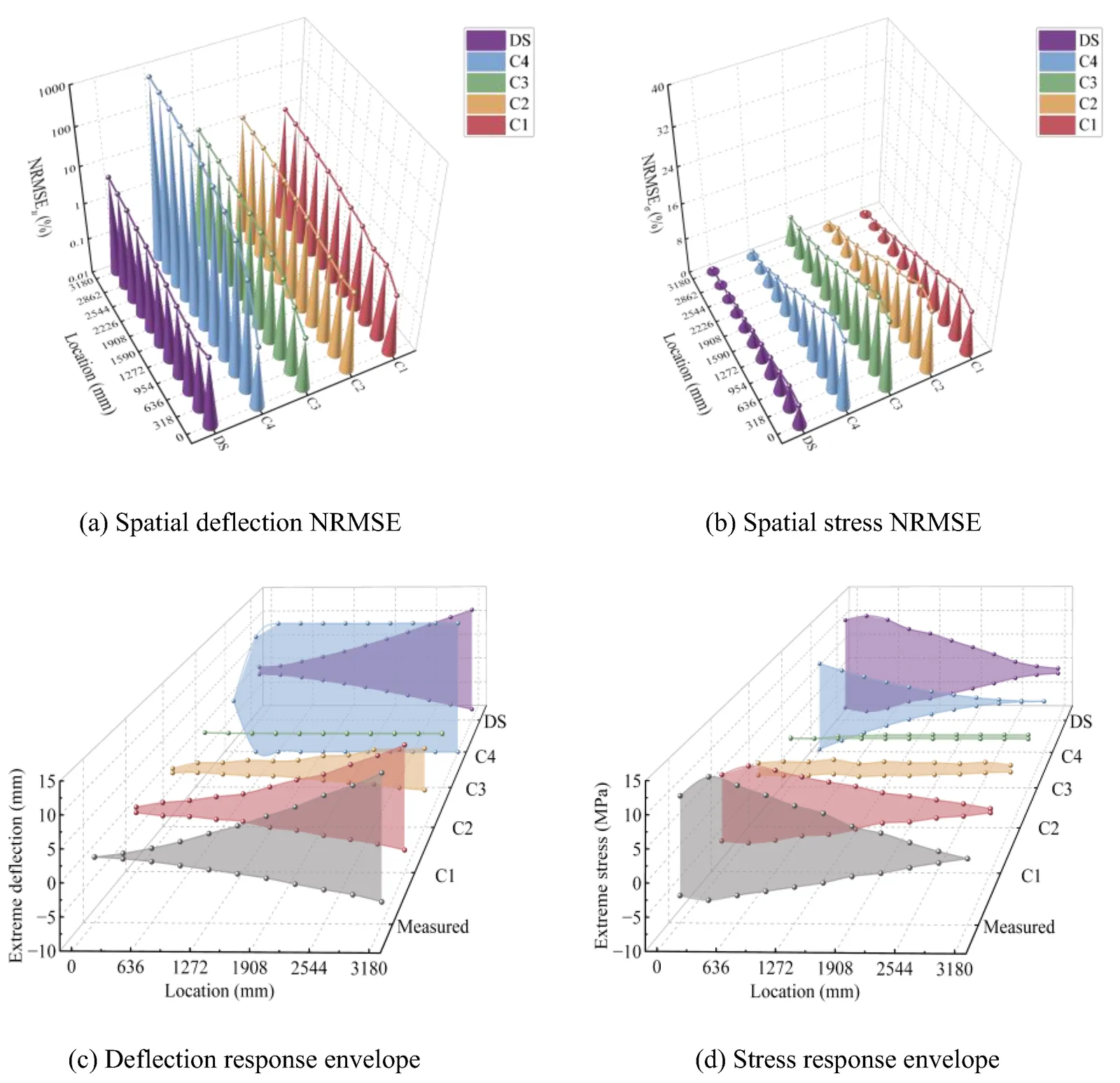
Spatial NRMSE distributions and response envelopes of benchmark models.

Figure 19 shows the spatial NRMSE and response envelopes. The spatial NRMSE of DS ranged from 0.75% to 5.04% for deflection, and its maximum stress NRMSE was 5.35%. Errors therefore remained low at every evaluation location. C1 retained the principal spatial pattern but showed biased envelope amplitudes. The envelopes of C2 and C3 contracted markedly, with C3 approaching a low-amplitude plane. The deflection envelope of C4 expanded anomalously, and its stress envelope also differed substantially from the measurements. DS thus maintained low spatial NRMSE across all evaluation locations and accurately reconstructed the spatial variation of deflection and stress response envelopes.

Simulation pretraining and experimental fine-tuning of DS-PINN took 19,366.5 s and 1476.2 s, respectively, for a total training time of 20,842.7 s excluding simulation data generation. C1 and C2 took 241.0 s and 157.7 s to train, respectively. Recorded inference times across the 4 test records were 0.7039 ± 0.0261 s for DS-PINN, 0.0234 ± 0.0091 s for C1, 0.0426 ± 0.0447 s for C2, 0.0949 ± 0.1385 s for C3, and 2.4995 ± 0.1708 s for C4. These values are means ± sample standard deviations of the per-record times obtained with each model’s inference procedure.

Under the present data and training conditions, DS-PINN provided a comparatively balanced performance across waveform correlation, overall error, extrema, and spatial distribution. These results reflect the combined contributions of spatiotemporal decoupling, simulation pretraining, and physical-domain adaptation to multi-location reconstruction from sparse measurements.

## 5. Conclusions

This study developed DS-PINN for multi-location reconstruction of low-frequency deflection and stress responses from sparse deflection measurements and limited experimental data. The results support four main conclusions.

(1) DS-PINN separates latent-space temporal evolution from continuous spatial decoding. Shared latent coordinates are combined with response-specific spatial bases for deflection and stress. This design avoids requiring a single coordinate network to evaluate temporal and spatial derivatives of different orders.

(2) Simulation pretraining provided an initial representation of the response fields, while physical-domain fine-tuning corrected temporal and amplitude differences between simulated and measured data. Across four independent 55 s test records, DS-PINN achieved PCC values of 0.9323 and 0.9093 for deflection and stress, respectively. The corresponding NRMSE values were 7.94% and 7.59%, while spatial NRMSE remained below 6% at all 11 locations.

(3) The ablation tests showed that the model components served distinct functions. Replacing the Newmark operator with an LSTM substantially reduced reconstruction accuracy. Causal convolution during fine-tuning widened the error distributions, whereas fixing the linear adapters compressed the response amplitudes. Removing the spatial-basis consistency constraint increased local oscillations in the spatial response profiles, although its effect on the overall metrics was small.

(4) Across the four test records, the mean deflection and stress NRMSE values for DS-PINN were approximately 45.0% and 45.9% lower, respectively, than those of the TCN baseline.

These results suggest that simulation-assisted virtual sensing has potential for estimating low-frequency deflection and stress responses from sparse deflection measurements and limited experimental data. The experimental evaluation covered 4 test records and 11 evaluation locations on a single beam. Assessing broader generalizability will require further validation with more records, denser spatial measurements and additional structural and loading configurations.

## Figures and Tables

**Table 1:** Geometric and material parameters of the beam.

Parameters	Symbol	Value	Unit
Beam length	*L*	3180	mm
Section width	*b*	101	mm
Section height	*h*	51	mm
Wall thickness	*t*	3	mm
Elastic modulus	*E*	2.1 × 10^5^	MPa
Poisson’s ratio	*ν*	0.3	-
Standard density	*ρ*_0_	7850	kg/m^3^

**Table 2:** Configurations of ablation variants.

Model	Configuration
A1	Replaced Newmark integration with a two-layer LSTM of width 64; increased the SIREN hidden width to 256; excluded the latent energy loss from pretraining Stage 3
A2	Removed the spatial-basis consistency loss ℒ_spa_
A3	Used causal convolution in place of the noncausal temporal extractor during fine-tuning
A4	Kept the linear adapters fixed during fine-tuning
DS	Complete DS-PINN

**Table 3:** Configurations of benchmark models.

Model	Benchmark Configuration
C1	TCN with 6 residual blocks and kernel size 3
C2	Transformer [34] with 4 encoder layers of dimension 128 and 8 attention heads
C3	PINN with 6 hidden layers, 256 units per layer and tanh activation
C4	PSO-calibrated analytical beam model with 8 modes and 7 adjustable parameters
DS	Complete DS-PINN

## Data Availability

The original contributions presented in this study are included in the article. Further inquiries can be directed to the corresponding author.
